# Biochemical and physiological characterization of *fut4* and *fut6* mutants defective in arabinogalactan-protein fucosylation in *Arabidopsis*

**DOI:** 10.1093/jxb/ert321

**Published:** 2013-10-14

**Authors:** Yan Liang, Debarati Basu, Sivakumar Pattathil, Wen-liang Xu, Alexandra Venetos, Stanton L. Martin, Ahmed Faik, Michael G. Hahn, Allan M. Showalter

**Affiliations:** ^1^Department of Environmental and Plant Biology, Ohio University, Athens, OH 45701, USA; ^2^Molecular and Cellular Biology Program, Ohio University, Athens, OH 45701, USA; ^3^Complex Carbohydrate Research Center, University of Georgia, Athens, GA 30602, USA; ^4^SAS Institute, 100 SAS Campus Drive, Cary, NC 27513, USA; ^5^Department of Plant Biology, University of Georgia, Athens, GA 30602, USA; ^6^ Present address: Joint BioEnergy Institute, 1 Cyclotron Rd. MS: 978-4121, Berkeley, CA 94720, USA; ^7^ Present address: Hua Zhong Normal University, Wuhan, Hubei 430079, China

**Keywords:** *Arabidopsis*, arabinogalactan-proteins, fucosyltransferase, glycosylation, hydroxyproline-rich proteins, plant cell wall.

## Abstract

Arabinogalactan-proteins (AGPs) are highly glycosylated hydroxyproline-rich glycoproteins present in plant cell walls. AGPs are characterized by arabinose-/galactose-rich side chains, which define their interactive molecular surface. Fucose residues are found in some dicotyledon AGPs, and AGP fucosylation is developmentally regulated. We previously identified *Arabidopsis thaliana FUT4* and *FUT6* genes as AGP-specific fucosyltransferases (FUTs) based on their enzymatic activities when heterologously expressed in tobacco (*Nicotiana tabacum*) BY2 suspension-cultured cells. Here, the functions of *FUT4* and *FUT6* and the physiological roles of fucosylated AGPs were further investigated using *Arabidopsis fut4*, *fut6*, and *fut4*/*fut6* mutant plants. All mutant plants showed no phenotypic differences compared to wild-type plants under physiological conditions, but showed reduced root growth in the presence of elevated NaCl. However, roots of wild-type and *fut4* mutant plants contained terminal fucose epitopes, which were absent in *fut6* and *fut4*/*fut6* mutant plants as indicated by eel lectin staining. Monosaccharide analysis showed fucose was present in wild-type leaf and root AGPs, but absent in *fut4* leaf AGPs and in *fut4*/*fut6* double mutant leaf and root AGPs, indicating that *FUT4* was required for fucosylation of leaf AGPs while both *FUT4* and *FUT6* contributed to fucosylation of root AGPs. Glycome profiling of cell wall fractions from mutant roots and leaves showed distinct glycome profiles compared to wild-type plants, indicating that fucosyl residues on AGPs may regulate intermolecular interactions between AGPs and other wall components. The current work exemplifies the possibilities of refinement of cell wall structures by manipulation of a single or a few cell wall biosynthetic genes.

## Introduction

Primary plant cell walls are a composite of complex carbohydrates and protein components. In dicotyledons, cellulose microfibres cross-linked by xyloglucan hemicellulose are thought to constitute the load-bearing framework, which is believed to be embedded in a matrix of pectic polymers made of homogalacturonan (HG), rhamnogalacturonan I (RG-I), rhamnogalacturonan II (RG-II), and protein components (Carpita and Gibeaut, 1993). The hydroxyproline-rich glycoprotein (HRGP) family represents a major group of plant cell wall proteins. The three subfamilies of HRGP—proline-rich proteins, extensins, and arabinogalactan-proteins (AGPs)—share the common feature of having hydroxyproline residues in their protein backbones and undergo glycosylation to various extents (Showalter, 1993; Shpak *et al.*, 2001). Proline-rich proteins are the least glycosylated and suggested to insert into and stabilize mature wall structure (Carpita and McCann, 2000). Extensins are moderately glycosylated, characterized by motifs for intra- and inter-molecular cross-linking, and are active players for wall self-assembly and plant defence (Cannon *et al.*, 2008; Lamport *et al.*, 2011; Memelink *et al.*, 1993; Wei and Shirsat, 2006). AGPs, having the greatest number of family members and the highest level of glycosylation of all the HRGPs, are implicated in various aspects of plant growth and development (Ellis *et al.*, 2010; Nothnagel, 1997; Seifert and Roberts, 2007; Showalter, 2001). As their name implies, AGPs are extensively glycosylated with type II arabinogalactan (AG) polysaccharides, which are mainly composed of galactose (Gal) and arabinose (Ara) residues, but may also contain other sugars, including rhamnose, glucuronic acid, galacturonic acid, and fucose (Fuc) (Ellis *et al.*, 2010; Nothnagel, 1997; Tan *et al.*, 2004). Given that the sugar side chains typically account for more than 90% of the molecular mass of AGPs, they are likely to define the interactive surface of the molecule and hence its function.

Recently, 85 AGP genes were identified in *Arabidopsis thaliana* using bioinformatics (Showalter *et al.*, 2010). However, the precise functions and mechanisms of action of most AGPs remain elusive. To address the function and regulation of AGPs it is necessary to understand the mechanism underlying AGP glycosylation. Although several glycosyltransferase activities were reported in *in vitro* assays for AGP glycosylation (Hayashi and Maclachlan, 1984; Liang *et al.*, 2010; Mascara and Fincher, 1982; Misawa *et al.*, 1996; Oka *et al.*, 2010; Schibeci *et al.*, 1984), to date there are only two fucosyltransferases genes (*FUT4* and *FUT6*) and two galactosyltransferase genes (*GALT2* and *GALT31A*) identified to encode glycosyltransferases specific for AGPs (Basu *et al.*, 2013; Geshi *et al.*, 2013; Wu *et al.*, 2010). In addition, Gille *et al.* (2013) identified a putative AGP β-arabinosyltransferase, in carbohydrate-active enzymes (CAZy) glycosyltransferase (GT) family 77 (CAZy GT77), based on genetic mutant analysis, although this finding is puzzling given that arabinose exists as an α-linked sugar in AGPs.

FUT4 and FUT6 belong to the CAZy GT37 family (http://www.cazy.org/GlycosylTransferases.html, last accessed 20 September 2013), the members of which are thought to encode α-1,2-FUTs (Sarria *et al.*, 2001), based on the characterized function of FUT1, which specifically fucosylates xyloglucans in pea and *Arabidopsis* (Faik *et al.*, 2000; Perrin *et al.*, 1999). AGPs with terminal Fuc residues have mainly been studied in cruciferous plants (Hashimoto, 2000). Monosaccharide composition analysis of AGPs from different organs of radish plants showed that AGP fucosylation was organ-specific and developmentally regulated (Tsumuraya *et al*., 1984, 1987, 1988). In *Arabidopsis*, Fuc was reported to be present in root AGPs (van Hengel and Roberts, 2002) and leaf AGPs (Tryfona *et al.*, 2012).

Previously, we demonstrated that the *Arabidopsis* FUT4 and FUT6 proteins fucosylate AGPs in biochemical assays *in vitro* and in a tobacco BY2 expression system, but no *in planta* data were provided (Wu *et al.*, 2010). Although, van Hengel and Roberts (2002) have shown that reduction in Fuc in *Arabidopsis* roots affected their development, no direct link to the genes involved in AGPs fucosylation was demonstrated. Here we demonstrate that the *FUT4* and *FUT6* genes are responsible for AGP fucosylation in *Arabidopsis* plants. Specifically, we focus on the biochemical and physiological characterization of *Arabidopsis fut4* and *fut6* mutants, and *fut4*/*fut6* double mutants, to corroborate our previous findings and to obtain insight into the physiological functions of AGP fucosylation.

## Materials and methods

### Plant materials and growth conditions

Two mutant lines (*fut4*, SAIL_284_B05; *fut4-2*, SALK_125310) for the *FUT4* gene (At2g15390) and two mutant lines (*fut6*, SALK_099500; *fut6-2*, SALK_078357) for the *FUT6* gene (At1g14080) in *A. thaliana* were analysed in this study. All of the mutant lines and wild-type plants were of the Columbia-0 ecotype. Seeds of T-DNA insertion lines were obtained from the Arabidopsis Biological Resource Centre (http://abrc.osu.edu/, last accessed 20 September 2013) in Columbus, OH, USA. Plants were grown in soil for mutant screening, seed harvesting, and growth-stage phenotypic analysis. For root harvesting, plants were grown hydroponically in water supplemented with Dyna-gro All-Pro 7-7-7 (Greentrees Hydroponics, Vista, CA, USA). The *Arabidopsis* hydroponic growth system was as described previously (Gibeaut *et al.*, 1997). For the phenotypic analysis of root growth, plants were grown on Murashige and Skoog (MS) medium (Caisson Laboratories, North Logan, UT, USA) containing 0.5% sucrose and 1g/l Phytogel. All plants were grown under long-day conditions (16h of light/8h of dark, 22 °C, 55% humidity) in growth chambers or growth rooms.

### Mutant confirmation by PCR and RT-PCR

**Fig. 1.**
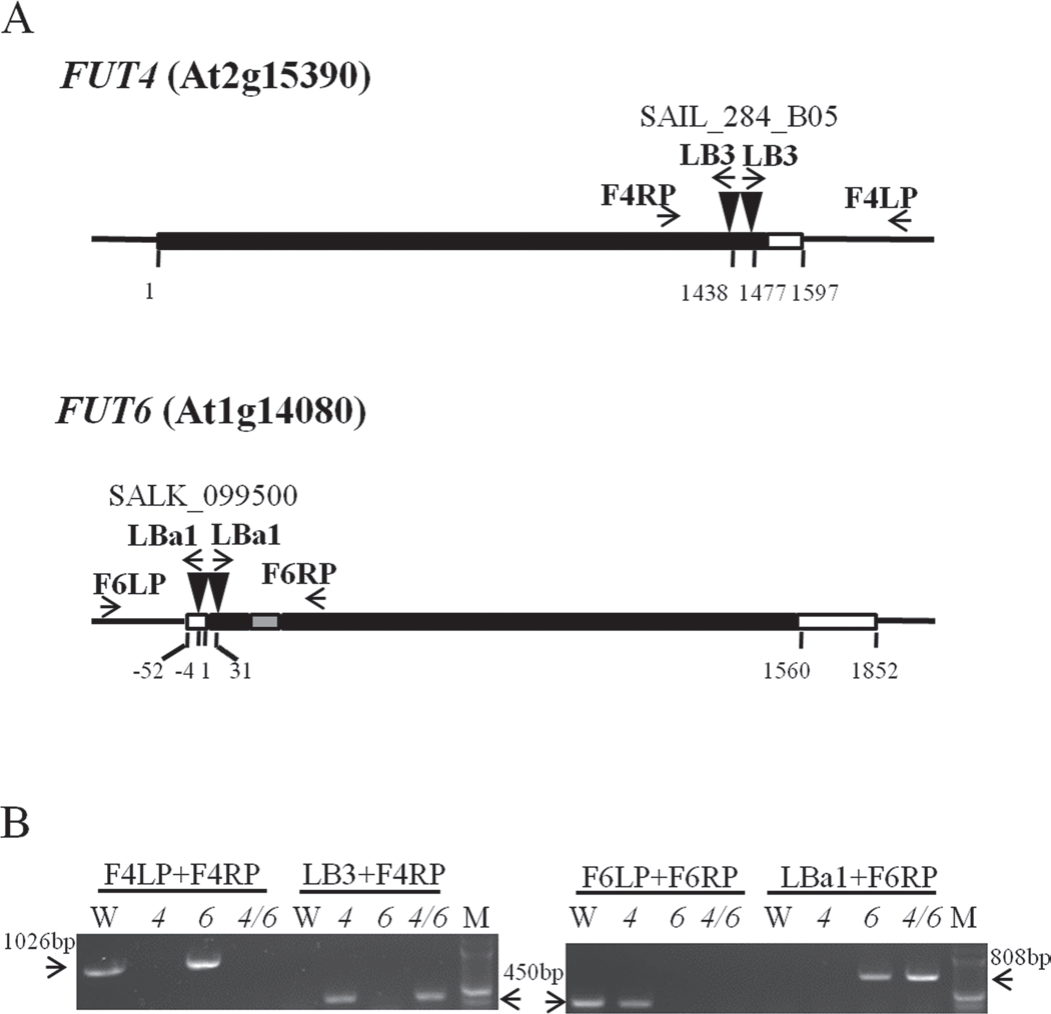
Identification of T-DNA insertion lines of *Arabidopsis fut4*, *fut6*, and *fut4*/*fut6* mutants by PCR. (A) Schematic diagrams of mutant *FUT4* and *FUT6* genes with T-DNA insertions. Black box, grey box, and white box represent exons, introns, and untranslated regions, respectively. Both the *fut4* (SAIL_284_B05) and *fut6* (SALK_099500) lines were identified to contain double T-DNA insertions (black triangles), as discussed in the text. (B) Genotyping of wild-type (W), *fut4* (*4*), *fut6* (*6*), and *fut4*/*fut6* (*4*/*6*) double mutant plants by PCR. Positions of the PCR primers are shown in (A) with arrows. The expected sizes amplified with primer pairs of F4LP and F4RP (1026bp), LB3 and F4RP (450bp), F6LP and F6RP (450bp), and LBa1 and F6RP (808bp) are indicated with arrows in (B). The 2-Log DNA ladder (New England Biolabs) was used as a molecular weight marker (M).

Genomic DNA isolation from *fut4*, *fut6*, and *fut4*/*fut6* mutant leaves and subsequent PCR analysis was carried out using Extract-N-Amp™ Plant Kits (Sigma-Aldrich, St Louis, MO, USA). The primer locations are indicated in Fig. 1, and the corresponding primer sequences are listed in Table S1. For sequencing purposes, PCR products were purified by gel extraction with QIAquick Gel Extraction Kit (Qiagen, Hilden, Germany) and sequenced by the Ohio University Genomics Facility (http://www.dna.ohio.edu/, last accessed 20 September 2013). To analyse transcript levels of *FUT4* and *FUT6*, total RNA was isolated from seedlings of wild-type and mutant plants 15 days after germination (DAG) using the RNeasy Plant Mini Kit (Qiagen) and the RNase-Free DNase Set (Qiagen). First-strand cDNA synthesis was performed from 2 μg of total RNA using oligo-dT (IDT) and Go Script reverse transcriptase (Promega, Madison, WI, USA). RT-PCR was performed using OneTaq DNA polymerase (New England Biolabs, Ipswich, MA, USA) and gene-specific primers (Table S1). The number of amplification cycles was 28 to evaluate and quantify differences among transcript levels before the reaction reached saturation.

For real-time quantitative PCR (qPCR) the cDNAs were amplified using Brilliant II SYBR Green QRT-PCR Master Mix with ROX (Agilent Technologies, La Jolla, CA, USA) in an MX3000P real-time PCR instrument (Agilent Technologies). PCR was optimized and reactions were performed in triplicate. The transcript level was standardized based on cDNA amplification of ubiquitin 10 (At4g05320) RNA as a reference. The F4rtF2+F4rtR2 primer pair was used for qPCR for *FUT4.* The F6rtF2+F6rtR2 primer pair was used for real-time qPCR for *FUT6.* Primer sequences are listed in Table S1. Relative gene expression data were generated using the wild-type plants as a calibrator.

### Phenotypic analysis

A growth-stage-based phenotypic analysis method was adopted from Boyes *et al.* (2001). Plant growth parameters including rosette perimeters (at 29 DAG), plant height (at 43 DAG), branch numbers (at 43 DAG), and plant weight (at 49 DAG) were measured and compared among wild-type, *fut4* and *fut6* mutant, and *fut4*/*fut6* double mutant plants (15 plants for each line). For the measurement of germination rate, mutant and wild-type seeds (over 50 seeds for each line) were sown on the same MS plate. Germinated seeds were counted under a light microscope every 12h after sowing the seeds. Statistical analysis was performed using the two-sample independent *t* test for continuous variables obtained from open source software, OpenEpi (http://www.openepi.com/OE2.3/Menu/OpenEpiMenu.htm, last accessed 20 September 2013).

For root morphology analysis, mutant and wild-type plants were grown on MS plates supplemented with 0.5% sucrose (over 24 plants for each line). The lengths of primary roots were recorded every 24h from 3 to 10 DAG, when the primary roots reach the edge of the petri plate. To test the response of the mutant lines to salt stress, wild-type and *fut* mutant lines were sown on NaCl-free MS plates supplemented with 0.5% sucrose and then transferred to MS plates supplemented with 3% sucrose and 150mM NaCl at 3 DAG. High sucrose (3%) is typically used in salt stress tests (Zhan *et al.*, 2012; Zhu *et al.*, 2007) to stimulate root elongation and allow easier detection of differences between genotypes. One-way analysis of variance (ANOVA) was performed using JMP software (SAS Institute, Cary, NC, USA) and showed a significant difference (*P*<0.0001) in root lengths among wild-type and mutant lines grown under salt stress. Subsequently, the Tukey honestly significant difference test was conducted to identify significance levels of differences in root lengths (Table S2).

### Eel lectin staining

Roots were harvested from 2-week-old wild-type, *fut4*, *fut6*, and *fut4*/*fut6* double mutant seedlings grown on MS plates. Freshly harvested roots were incubated in a solution of eel lectin conjugated to Texas Red (EY Laboratories, San Mateo, CA, USA) dissolved in 20 µg ml^−1^ in 10mM phosphate-buffered saline (pH 7.3) for 3h in the dark at room temperature. After incubation, roots were rinsed with 10mM phosphate-buffered saline (pH 7.3) and observed under a Motic BA400 EPI-Fluorescence Upright Biological Microscope (Motic Microscope, British Columbia, Canada) using a Texas Red/Cy3.5 filter set. Images were captured using Motic Images Plus 2.0 software (Motic Microscope).

### Monosaccharide composition analysis by gas chromatography-mass spectrometry (GC-MS)

AGPs were isolated from leaves and roots of wild type, *fut4*, *fut6*, and *fut4*/*fut6* double mutant plants as reported previously (Schultz *et al.*, 2000). AGP samples were quantified using a Yariv precipitation method (Xu *et al.*, 2008) and gum arabic as standards. Samples were analysed by both in-house GC-MS instruments at Ohio University and by analytical services at the Complex Carbohydrate Research Center (CCRC) at the University of Georgia (http://www.ccrc.uga.edu/, last accessed 20 September 2013). Briefly, 100 µg AGP samples were hydrolysed in 2M trifluoroacetic acid (2h in sealed tube at 121 °C), reduced with NaBD_4_, and acetylated using acetic anhydride/trifluoroacetic acid. Inositol, 20 µg, was added as an internal standard to all samples. For the in-house GC-MS system, alditol acetates were analysed on a Trace GC Ultra interfaced to a DSQII mass spectrometer (Thermo Scientific, Waltham, MA, USA). Separation was performed on a 30 m FactorFOUR VF-23ms capillary column (Varian, Palo Alto, CA, USA). At the CCRC, alditol acetates were analysed on a 7890A GC interfaced to a 5975C mass spectrometer (Agilent Technologies). Separation was performed on a 30 m Supelco 2330 capillary column (Sigma-Aldrich).

### Monoclonal antibodies

Monoclonal antibodies were obtained as hybridoma cell culture supernatants from laboratory stocks at the CCRC [available from CarboSource Services (http://www.carbosource.net, last accessed 20 September 2013)]. A detailed list of the antibodies used, grouped according to the polysaccharide primarily recognized by the antibodies (Pattathil *et al.* 2010), is provided in Table S3, which also includes links to a database, Wall*Mab*DB (http://www.wallmabdb.net, last accessed 20 September 2013), containing more detailed information about each antibody.

### Preparation of alcohol-insoluble residue extracts and fractionation

Plant tissues were isolated and ground to a fine powder using liquid nitrogen and a mortar and pestle. The powder was then suspended in 80% (v/v) ethanol, vortexed, and centrifuged at 3000 *g* for 5min. The residue was then suspended in absolute ethanol and centrifuged as above. Subsequently, the residue was suspended in chloroform/methanol (1:1, v/v) and stirred for 1h at room temperature. This suspension was centrifuged again and the alcohol-insoluble residue extract was washed with acetone. All the above steps were repeated, and the final residue was air-dried at room temperature.

Alcohol-insoluble residue fractionation was done to obtain an overall picture of the glycan epitope composition of the walls and gain insight into how tightly these epitopes are bound to the wall matrix. For this purpose, alcohol-insoluble residue samples were sequentially extracted with 50mM ammonium oxalate, pH 5.0, 50mM sodium carbonate, pH 10 (containing 0.5%, w/v, sodium borohydride), 1M KOH (containing 1%, w/v, sodium borohydride), and 4M KOH (containing 1%, w/v, sodium borohydride). Each treatment was done for a period of 24h, after which time the samples were centrifuged at 4000 *g* for 15min and the supernatants decanted. The pellets were washed with water and centrifuged as before; the water wash was discarded and the pellet retained for the next extraction. Each wall extract was neutralized (if necessary), dialysed extensively against water, and lyophilized for further analysis. Detailed protocols for these analyses can be found in Pattathil *et al.* (2012).

### Total sugar estimation and enzyme-linked immunosorbent assay (ELISA)

Cell wall extracts were dissolved in de-ionized water (0.2mg ml^−1^) and total sugar contents of cell wall extracts were estimated using the phenol/sulphuric acid method (DuBois *et al.*, 1956; Masuko *et al.*, 2005). All solubilized fractions were adjusted to an equal amount of total carbohydrate content prior to ELISA assay. Cell wall extracts (60 μg sugar ml^−1^) were applied to the wells of 96-well ELISA plates (Costar 3598) at 50 μl per well and allowed to evaporate to dryness overnight at 37 °C. A Biotek robotic system (Biotek, Winooski, VT, USA) was used to perform fully automated ELISAs with a series of 152 monoclonal antibodies directed against diverse plant cell wall carbohydrate epitopes (Pattathil *et al.*, 2012). ELISA data are presented as heat maps (glycome profiles) in which the antibody order and groupings are based on a hierarchical clustering analysis that groups the antibodies based on similarities in their binding patterns to a panel of diverse plant glycans (Pattathil *et al.*, 2010).

### Statistical analysis of glycome-profiling data

Data was analysed using JMP Genomics 6.1 (SAS Institute). Distribution analysis, principal variance components analysis, and ANOVA were performed on the both raw and mean normalized data. For the principal variance components and ANOVA analyses, the plant genotype (wild type, *fut4*, *fut6*, or *fut4*/*fut6*) and the fraction (oxalate, carbonate, 1M KOH, or 4M KOH) were considered to be fixed effects while the replicates were considered random effects. A false discovery rate with an alpha level of 0.05 was used to correct for multiple comparisons.

### Expression analysis of *FUT4* and *FUT6* genes based on microarray data from Genevestigator

Expression data for the *FUT4* and *FUT6* genes were acquired from the Genevestigator website (https://www.genevestigator.com/gv/, last accessed 20 September 2013) using the ‘perturbations’ condition search. Filter options were used to select for fold changes in expression levels (>2 or <−2) and for *P*<0.05.

## Results

### Isolation of T-DNA insertion lines of *fut4* and *fut6* and generation of the *fut4*/*fut6* double mutant

**Fig. 2.**
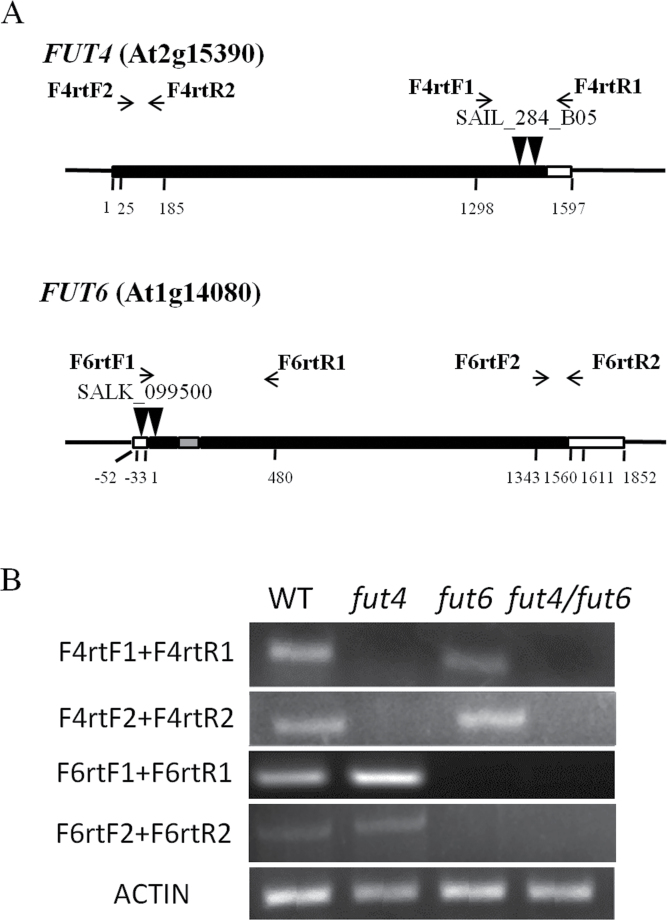
RNA transcript levels of *FUT4* and *FUT6* genes in homozygous *fut4* and *fut6* mutants, *fut4*/*fut6* double mutants, and wild-type (WT) seedlings. (A) Schematic diagrams of mutant *FUT4* and *FUT6* genes with T-DNA insertions. Black box, grey box, and white box represent exons, introns, and untranslated regions, respectively. (B) RT-PCR of total RNAs isolated from *Arabidopsis* seedlings 15 DAG. Positions of the RT-PCR primers are shown in (A) with arrows. *ACTIN* was used as a control for loading.

*Arabidopsis fut4* (SAIL_284_B05) and *fut6* (SALK_099500) single mutants with T-DNA insertions in the exons of *FUT4* or *FUT6* genes were ordered from the Arabidopsis Biological Resource Center and used to obtain homozygous mutants. Genotyping of the homozygous mutant plants was performed by PCR analysis using wild-type controls (Fig. 1). Flanking regions of left borders of the T-DNA insertions were amplified and sequenced with T-DNA left border (LB) primers and *FUT4*- or *FUT6*-specific right primers (RP). DNA sequencing showed that the insertion sites were located at nucleotide position 1438 (relative to the first nucleotide of the start codon designated as position 1) for *fut4* and nucleotide position 31 for *fut6*, which were consistent with the records of insertion sites of the mutant lines on the Salk Institute Genomic Analysis Laboratory website (SIGnAL; http://signal.salk.edu/cgi-bin/tdnaexpress, last accessed 20 September 2013). To confirm that the flanking regions of the right borders of the T-DNA insertions were also restricted to the *fut4* and *fut6* genes, PCRs with T-DNA right border (RB) primers and *FUT4*- or *FUT6*-specific left primers (LP) were performed, but no amplifications were obtained. Instead, specific bands were amplified using primer pairs of LB and LP (data not shown). Sequencing results of the product amplified with LB and LP indicated that a second T-DNA insertion was located at nucleotide position 1477 in *fut4*, downstream and in the opposite direction of the insertion at position 1438 (Fig. 1). Similarly in *fut6*, sequencing showed that at least two T-DNA insertions, in opposite directions, interrupted *FUT6* between nucleotide position −4 and position 31. The occurrence of such T-DNA insertions as tandem repeats is not infrequent (Ponce *et al.*, 1998). Absence of the *FUT4* and *FUT6* transcripts in the corresponding single mutant lines was confirmed by RT-PCR (Fig. 2) and real-time qPCR (Fig. S1). Although by RT-PCR little change in the expression of the *FUT4* and *FUT6* genes in mutant lines compared to wild type was observed, real-time qPCR showed that *FUT4* expression increased by 4-fold in leaf tissues of the *fut6* mutant.

Homozygous *fut4* and *fut6* single mutants were crossed, and the F_1_ generation was self-crossed before screening the F_2_ generation to obtain homozygous *fut4*/*fut6* double mutants (Fig. 1). As expected, no *FUT4* or *FUT6* transcripts were observed in the double mutant line (Fig. 2 and Fig. S1).

### Phenotypic analysis of *fut4*, *fut6*, and *fut4*/*fut6* mutants under physiological growth conditions

**Fig. 3.**
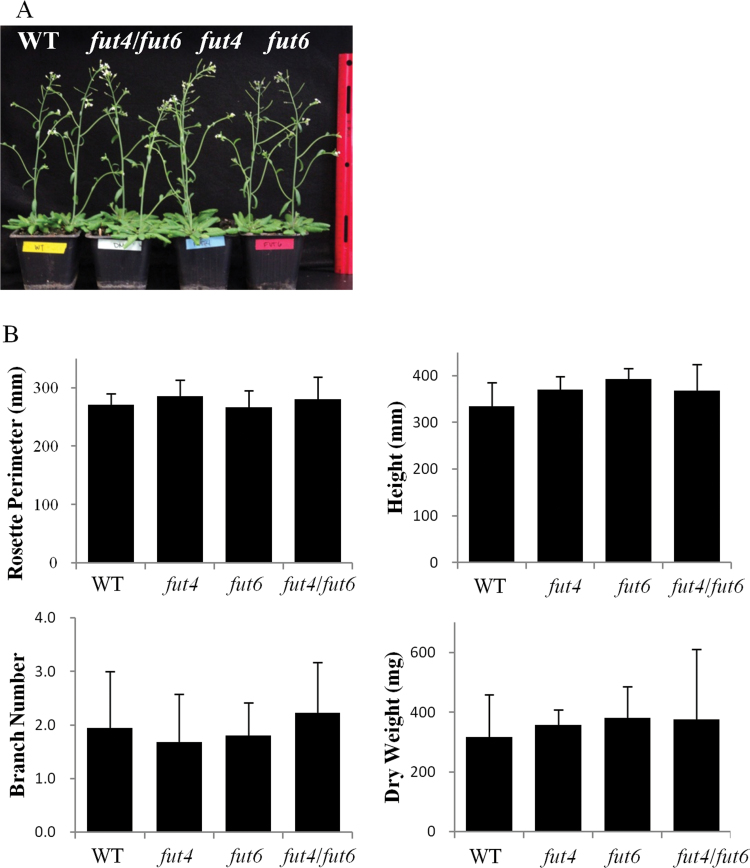
Phenotypic analysis of *Arabidopsis fut4*, *fut6*, and *fut4*/*fut6* mutant plants compared to wild-type (WT) plants under physiological growth conditions. (A) Whole plant images of wild type, *fut4*, *fut6*, and *fut4*/*fut6* mutant plants grown in soil at 32 DAG. (B) The rosette perimeters (at 29 DAG), plant height (at 43 DAG), branch numbers (at 43 DAG), and plant dry weight (at 49 DAG) compared among wild-type and *fut4*, *fut6*, and *fut4*/*fut6* mutant plants. Data and error bars represent mean±SD (*n*=15). No significant differences were identified between wild type and any of the mutant lines for the parameters measured, as indicated by *P*>0.05 in Student’s *t* tests for continuous variables.

Growth-stage-based phenotypic analysis (Boyes *et al.*, 2001) was performed to evaluate growth and reproduction of the *fut4*, *fut6*, and *fut4*/*fut6* mutants compared to wild-type *Arabidopsis* plants. However, no significant differences were observed among the *fut4*, *fut6*, and *fut4*/*fut6* mutants and wild-type plants in terms of rosette size, plant height, dry weight, and stem branching numbers (Fig. 3). The *fut4*, *fut6*, and *fut4*/*fut6* mutants did not show abnormalities in reproduction as indicated by similar flowering times, seed yields, and seed germination rates compared to wild-type plants under physiological growth conditions (Table 1 and Fig. S2).

**Table 1.** Flowering time and silique numbers of wild type, fut4, fut6, and fut4/fut6 mutant plants grown in soil under physiological growth conditions

	Wild type	*fut4*	*fut6*	*fut4*/*fut6*
Flowering time (days±SD)^a^	24.3±0.7	24.0±0.0	24.0±0.0	24.2±0.6
Number of siliques (number per plant±SD)^b^	181±71	221±50	229±69	224±118

^a^Flowering time was recorded when the first flower buds were visible.

^b^Number of siliques was counted at 49 DAG (*n*=15). No significant differences were identified between wild type and any of the mutant lines for flowering time and number of siliques, as indicated by *P*>0.05 in Student’s *t* tests for continuous variables.

**Fig. 4.**
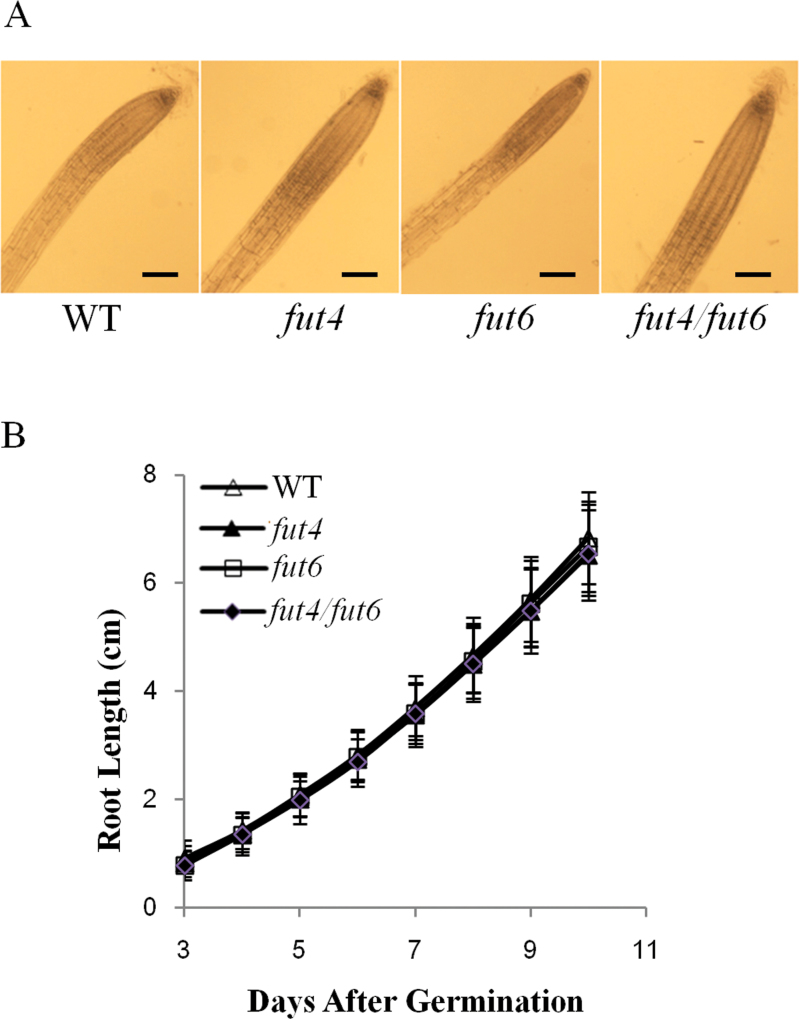
Root growth of *Arabidopsis* wild-type (WT) and *fut4*, *fut6*, and *fut4*/*fut6* mutant plants. Plants were grown on MS plates containing 0.5% sucrose. (A) Light-microscope images of wild-type, *fut4*, *fut6*, and *fut4*/*fut6* roots at 15 DAG. Scale bars, 10 µm. (B) Root length was measured daily as a function of time from 3 to 10 DAG. Values shown were means of data from over 24 individual plants per line, with standard errors shown as error bars.

To examine root growth of the mutant plants, *fut4*, *fut6*, and *fut4*/*fut6* mutants and wild-type plants were grown on media plates containing MS salts supplemented with 0.5% sucrose. No significant differences in root morphology or root growth rates were observed among wild type and *fut4*, *fut6*, and *fut4*/*fut6* mutant plants (Fig. 4). In addition, transverse sections of roots from wild-type and *fut4*/*fut6* double mutant plants were stained with toluidine blue and observed under a light microscope. No detectable differences were found (data not shown).

### *fut4*, *fut6*, and *fut4*/*fut6* mutants show reduced root growth under salt stress

**Fig. 5.**
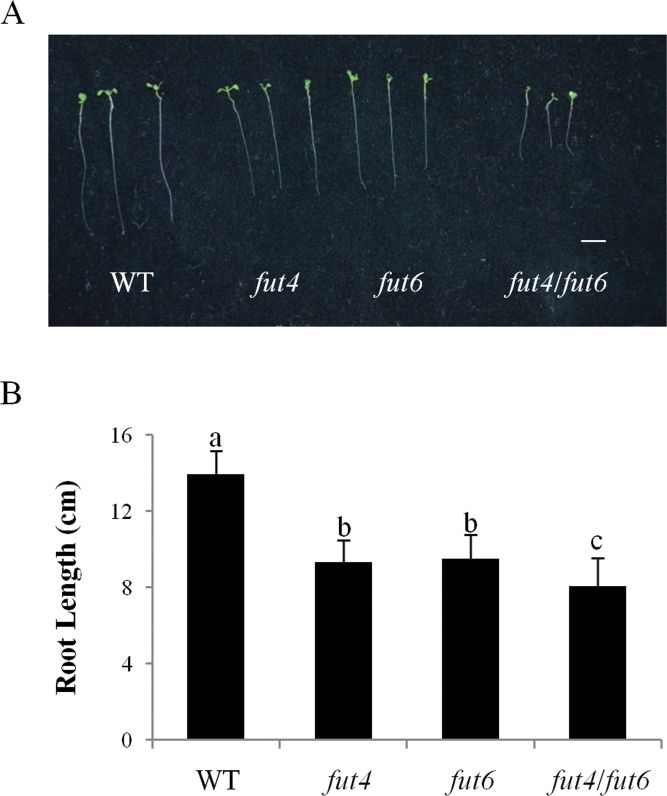
Root length measurement of *Arabidopsis fut4*, *fut6*, and *fut4*/*fut6* mutant plants and wild-type (WT) plants grown under salt stress. *Arabidopsis* seeds were germinated on NaCl-free MS plates and then transferred to MS plates containing 150mM NaCl at 3 DAG. (A) Seedling images of wild-type and *fut4*, *fut6*, and *fut4*/*fut6* mutant plants on the tenth day after transfer. Three representative seedlings of each plant line were photographed. Scale bar, 3cm. (B) Measurement of primary root length on the tenth day after transfer. Data and error bars represent mean±SD (*n*=31). Values annotated with different letters are significantly different (*P*<0.01; Tukey honestly significant difference test).

To test the effect of salt stress on root growth, *fut4*, *fut6*, and *fut4*/*fut6* mutants and wild-type plants were germinated on NaCl-free MS plates and then transferred to MS plates containing 150mM NaCl at 3 DAG. *fut4* and *fut6* mutants showed reduced root length compared to wild-type plants. In *fut4*/*fut6* double mutant plants the primary root length was further reduced compared to *fut4* and *fut6* single mutant lines (Fig. 5 and Table S2).

### Eel lectin shows a different staining pattern in roots of *fut6* and *fut4*/*fut6* mutant plants compared to *fut4* and wild-type plants

**Fig. 6.**
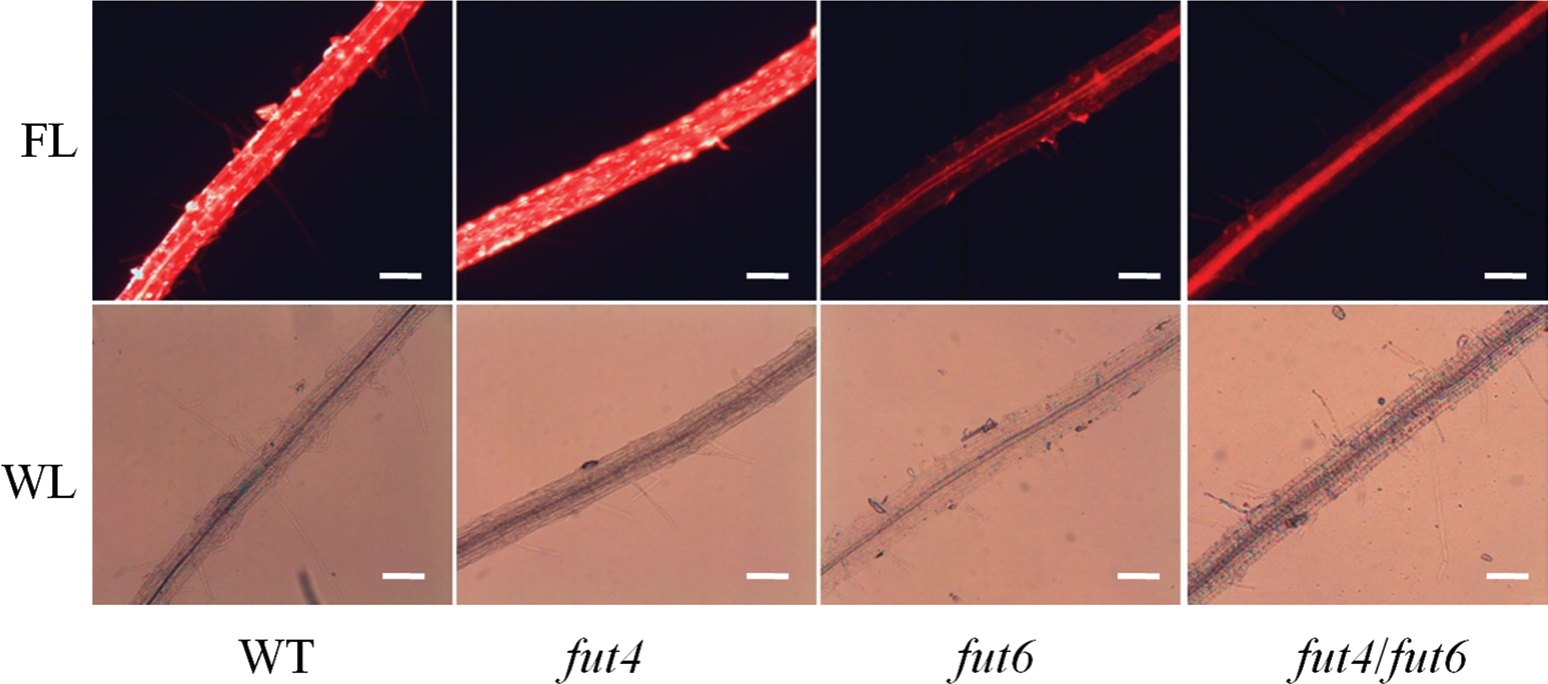
Eel lectin staining of roots from wild-type (WT) and *fut4*, *fut6*, and *fut4*/*fut6* mutant plants. Eel lectin conjugated to Texas Red was used to stain the roots of *Arabidopsis* plants grown on MS media containing 0.5% sucrose for 14 days. The stained roots were observed under fluorescent light (FL) and white light (WL) as indicated. Scale bars, 10 µm.

Screening for and characterization of Fuc residues on AGPs from cruciferous plants were performed using eel lectin (Hashimoto, 2000), a reagent that binds specifically to terminal α-l-Fuc residues (Springer and Desai, 1971; Watkins and Morgan, 1952). van Hengel and Roberts (2002) showed that eel lectin recognizes α-l-Fuc attached to α-l-Ara residues in *Arabidopsis* root AGPs, but not α-l-Fuc-(1→2)-β-d-Gal in RG-I. The binding specificity of eel lectin is also different from the specificity of the monoclonal antibody, CCRC-M1, which recognizes the α-(1→2)-linked fucosyl epitope on xyloglucan (Puhlmann *et al.*, 1994; van Hengel and Roberts, 2002). When whole roots of 14-day-old *Arabidopsis* plants were subjected to staining with eel lectin conjugated to Texas Red, the wild-type and *fut4* mutant plants showed a bright, patchy staining pattern on the root surface, which was absent in the *fut6* and *fut4*/*fut6* mutant roots (Fig. 6). No observable differences were seen in the leaves from wild-type or *fut4*, *fut6*, and *fut4*/*fut6* mutants stained with eel lectin conjugated to Texas Red (data not shown).

### *fut4*/*fut6* mutants are defective in AGP fucosylation in roots and leaves

Because *FUT6* was mostly expressed in root while *FUT4* showed the greatest expression in leaf but was also expressed in root (Sarria *et al.*, 2001), leaf and root AGPs were purified using β-Yariv reagent from wild-type and *fut4*, *fut6*, and *fut4*/*fut6* mutant plants and subjected to monosaccharide composition analysis using GC-MS. As expected, the GC-MS data showed that AGPs from all samples were mainly composed of Gal and Ara residues, with a ratio of approximately 3:1 for root samples and approximately 2:1 for leaf samples in both wild-type and mutant lines with subtle variations (Table 2). Compared to wild type, both *fut4* and *fut6* showed a decrease of Fuc content by approximately 50% in root AGPs, while the *fut4*/*fut6* double mutant contained no detectable Fuc in their root AGPs. Fuc was also not detected in AGPs from *fut4*/*fut6* and *fut4* mutant leaves. In contrast, the Fuc content in leaf AGPs from *fut6* was increased by approximately 1.5-fold compared to the wild type. The increase of Fuc content in *fut6* leaves may be attributed to the increased expression of *FUT4* in compensating for the lost of *FUT6* expression as revealed by real-time qPCR (Fig. S1). It should be noted that monosaccharides were detected using the alditol acetate sugar derivatization method that cannot detect acidic sugars. Thus, although uronic acid sugars (i.e. glucuronic acid) are known to be present in *Arabidopsis* AGPs (Tryfona *et al.*, 2012; van Hengel and Roberts, 2002), they are not indicated in our data.

**Table 2.** *Neutral monosaccharide content of purified AGPs from roots and leaves of wild type*, fut4, fut6, *and* fut4/fut6 *mutant plants*

	Root AGPs				Leaf AGPs			
	Wild type	*fut4*	*fut6*	*fut4*/*fut6*	Wild type	*fut4*	*fut6*	*fut4*/*fut6*
Ara	23.8±2.4	23.2±1.0	24.9±2.5	26.4±2.0	34.3±1.1	38.1±1.3	35.8±0.4	39.2±0.6
Gal	70.6±1.0	70.1±1.9	73.0±0.5	72.0±0.1	61.6±1.1	61.9±1.3	61.6±1.1	60.8±0.6
Fuc	1.8±0.2	0.8±0.1	1.0±0.1	ND	2.1±0.1	ND	3.5±0.6	ND
Glc	2.2±0.2	5.9±0.8	1.1±1.0	2.3±1.6	ND	ND	ND	ND

AGP samples were derivatized by the alditol acetate method and analysed using GC-MS. The molar percentage of each sugar is presented. The values are averages of two biological replicates. The standard deviations are indicated. ND, not detected.

### Glycome profiling of root and leaf cell walls of wild-type and *fut4*, *fut6*, and *fut4*/*fut6* mutant plants

To examine alterations in the compositions and interactive properties of cell wall polysaccharides in *fut4*, *fut6*, and *fut4*/*fut6* mutants compared to wild-type plants, total cell walls from leaf and root materials were sequentially extracted and the solubilized glycans were subjected to glycome profiling. Glycome profiling provides both a qualitative and quantitative measure of cell wall glycan epitopes present in the walls using a high-throughput ELISA method (Pattathil *et al.*, 2012). Approximately 150 monoclonal antibodies, which recognize 19 groups of glycan epitopes present in most major classes of cell wall glycans, were used in the ELISAs. The output of these ELISAs is typically shown as a heat map, where the antibodies are grouped according to their reactivities with a diverse panel of plant cell wall glycans (Pattathil *et al.*, 2010).

**Fig. 7.**
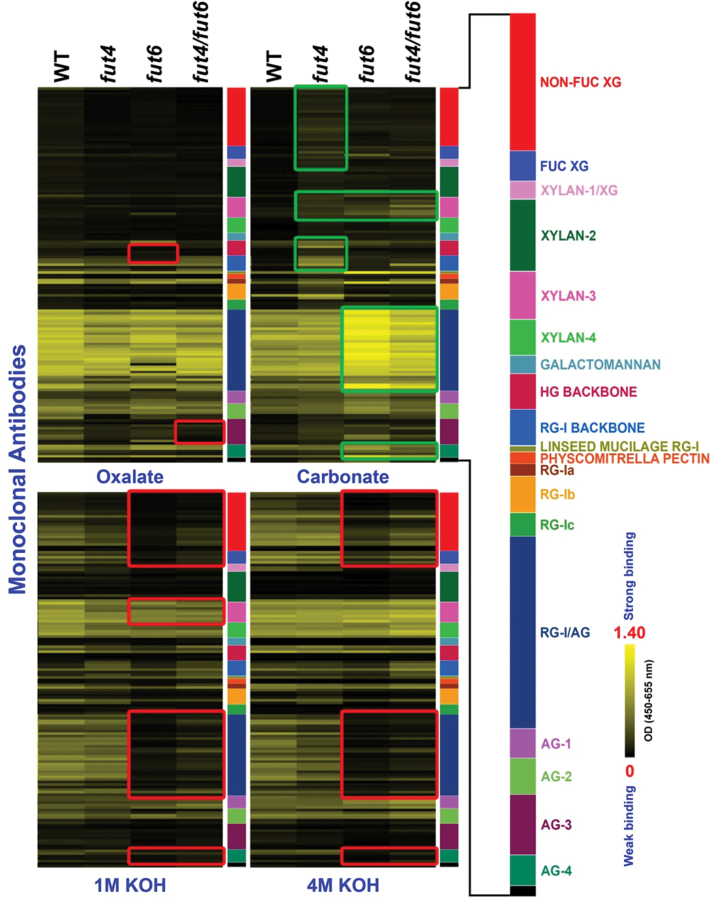
Glycome profiling of sequential cell wall extracts from roots of wild-type (WT) and *fut4*, *fut6*, and *fut4*/*fut6* mutant plants. The presence of cell wall glycan epitopes in each extract (as indicated at the bottom of each column) was determined by ELISAs using 152 glycan-directed monoclonal antibodies. The data are presented as heat maps, with bright yellow indicating strongest binding and black indicating no binding. The altered patterns of monoclonal antibody binding in the mutant fractions compared to wild-type fractions are highlighted by green (increased binding) or red (decreased binding) rectangles.

**Fig. 8.**
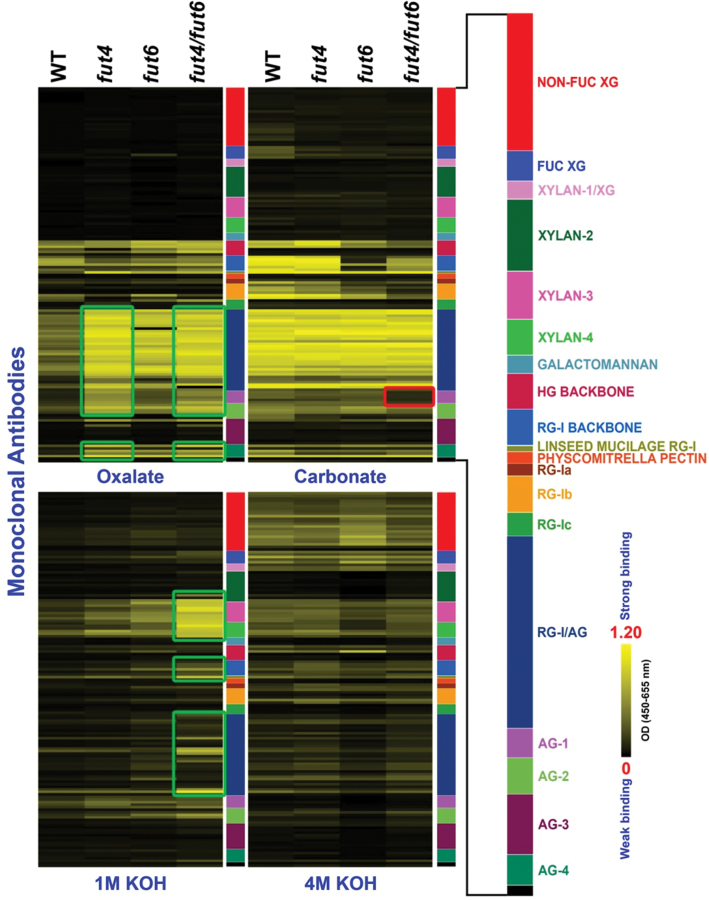
Glycome profiling of sequential cell wall extracts from leaves of wild-type (WT) and *fut4*, *fut6*, and *fut4*/*fut6* mutant plants. The presence of cell wall glycan epitopes in each extract (as indicated at the bottom of each column) was determined by ELISAs using 152 glycan-directed monoclonal antibodies. Details are as for Figure 7.

Glycome profiling of cell wall materials from roots and leaves of two biological replicates of plant material was performed. Because the glycome profiles obtained from the two biological replicates of plant material were almost identical, only one set of the glycome profiles is shown (Figs 7 and 8). Cell wall materials were subjected to four sequential extractions including an oxalate extraction, followed by alkaline extractions, with progressively increasing strengths. Oxalate binds to and depletes Ca^2+^ from cell walls, during and after which the most loosely associated pectic polysaccharides as well as AGPs are solubilized (Günter *et al.*, 2004; Gorshkova *et al.*, 1996). Alkali solutions of different strengths extract mainly hemicellulose (Fry, 1988), but also solubilize more tightly bound pectins and AGPs. For the wild-type root sample, consistent with the extraction method, pectic and AG epitopes, including epitopes for RG-I/AG and AG-1, 2, 3, and 4, started to be extracted by oxalate and carbonate solutions, while most epitopes for hemicelluloses, including epitopes for non-fucosylated xyloglucans (NON-FUC XG), fucosylated xyloglucans (FUC XG), and xylans (recognized by XYLAN-1, 2, 3, and 4 groups of antibodies) were extracted by the 1 and 4M KOH extracts (Fig. 7). It should be noted that fractionation by no means separates cell wall components into pure polymers (Fry, 1988) and that epitopes recognized by a given monoclonal antibody may be present on different wall polymers (Pattathil *et al.*, 2010).

Glycome profiles of cell wall extracts from roots of *fut4*, *fut6*, and *fut4*/*fut6* mutants were different to varying extents from the corresponding extracts prepared from wild-type roots (Fig. 7). In the oxalate extract, epitopes for the HG BACKBONE and AG-3 groups of antibodies showed decreased abundance in the *fut6* and *fut4*/*fut6* lines, respectively. The carbonate extract of *fut4* roots exhibited signals for hemicellulose and pectin backbone epitopes, i.e. NON-FUC XG, FUC XG, XYLAN-1, 3, HG BACKBONE, and RG-I BACKBONE, which were largely absent in the same extract of wild-type samples. The *fut6* and *fut4*/*fut6* lines showed decreased abundance for epitopes for NON-FUC XG and FUC XG in 1 and 4M KOH extracts compared to wild type. Interestingly, in the *fut6* and *fut4*/*fut6* lines, epitopes for RG-I/AG and AG-4 had decreased signal intensities in the 1 and 4M KOH extracts, but increased signal intensities in the carbonate extract. Similarly, in all three mutant lines, epitopes for XYLAN-3 showed reduced signal intensity in the 1M KOH extract, but epitope signal intensity increased in the carbonate extract.

Glycome profiles of leaves from *fut4*, *fut6*, *fut4*/*fut6* and wild-type plants showed similar patterns to one another in the carbonate and 4M KOH extracts (Fig. 8). In the oxalate extract, increased signals for pectic AG epitopes (RG-I/AG) were observed in all three mutant lines and increased AG epitopes (AG-1, 2, 4) were observed in the *fut4* and *fut4*/*fut6* mutant lines compared to the wild-type line. In addition, the 1M KOH extract in the *fut4*/*fut6* double mutant showed increased signals for multiple epitopes, including XYLAN-3, XYLAN-4, RG-I BACKBONE, LINSEED MUCILAGE RG-I, and RG-I/AG.

The observed differences in the glycome-profiling studies described above have been validated and confirmed by statistical analyses. The principal variance components analysis revealed that 95.9% of the observed variation in antibody binding within a given fraction was due to differences in the plant genotypes. ANOVA revealed changes very similar to the glycome profiles shown in Figures 7 and 8. The differences in antibody binding that were shown to be significantly different in the ANOVA are tabulated in Table S4.

### Analysis of allelic mutant lines of *fut4* and *fut6*

One allelic mutant line for *FUT4* (*fut4-2*, SALK_125310) and one allelic mutant line for *FUT6* (*fut6-2*, SALK_078357) were identified from SALK mutant collections from the Arabidopsis Biological Resource Centre. Homozygous *fut4-2* and *fut6-2* contain no *FUT4* or *FUT6* transcripts, respectively, as shown by RT-PCR analysis (Fig. S3). Real-time qPCR analysis showed that *FUT4* expression increased by 2.3- and 2.8-fold in root and leaf tissues of *fut6-2* mutant, respectively (Fig. S4). Similar to *fut4* and *fut6*, *fut4-2* and *fut6-2* have normal root growth on standard MS plates (Fig. S5). When grown on MS plates supplemented with 150mM NaCl the root lengths of *fut4-2* and *fut6-2* mutants were significantly shorter than in the wild type but longer than in the *fut4*/*fut6* double mutant (Fig. S6, Fig. 5, and Table S2).

## Discussion

Over 1000 *Arabidopsis* genes have been predicted to encode genes involved in cell wall biosynthesis (Yong *et al.*, 2005). However, to date only about two dozen genes have been identified unambiguously as GTs or glycan synthases responsible for the biosynthesis of specific cell wall molecules. Identification of cell wall biosynthetic enzymes through a biochemical purification route is often hampered by the low abundance of the enzymes or difficulties in maintaining enzymatic activities during the solubilization/purification process. Genetic strategies are also potentially hampered by functional redundancy of cell wall synthetic genes or the plasticity of cell wall structure, which is often insensitive to the disruption of single genes (Reiter *et al.*, 1997). Alternatively, bioinformatics provides a means to identify candidate genes involved in the biosynthesis of particular wall molecules, although additional research is required to verify the function of the genes identified in this manner. Using a bioinformatic strategy, eight *Arabidopsis* genes, namely *FUT2*–*FUT9* (Sarria *et al.*, 2001), were identified as homologues to *Arabidopsis FUT1*, which has a defined function as an α-1,2-FUT specific for xyloglucan fucosylation in all organs of *Arabidopsis* plants (Faik *et al.*, 2000; Perrin *et al.*, 1999). We previously demonstrated that the *FUT4* and *FUT6* genes are AGP FUTs by introducing the *FUT4* and *FUT6* genes into tobacco BY2 cells, which normally do not contain fucosylated AGPs, and showing that AGP FUT activities are acquired by the transgenic BY2 cells (Wu *et al.*, 2010). In this report, *fut4* and *fut6* null mutants as well as a *fut4*/*fut6* double mutant were utilized to corroborate our previous biochemical findings using molecular genetics and to obtain insight into the physiological functions of *FUT4* and *FUT6* in *Arabidopsis* plants.

Expression analysis has shown that *FUT6* is expressed nearly exclusively in the root with only trace expression in flowers, while *FUT4* is expressed more ubiquitously in root, stem, leaf, and flower and has the highest expression in leaf (Sarria *et al.*, 2001). Monosaccharide composition analyses of the AGPs isolated from the *fut* mutants examined in this study (Table 2) are consistent with these previous gene expression studies. Specifically, Fuc residues are present in wild-type root and leaf AGPs, but absent in root and leaf AGPs from the *fut4*/*fut6* mutant. Moreover, the *fut4* and *fut6* single mutants had reduced Fuc content in their root AGPs. Fuc residues were absent in *fut4* leaf AGPs, but present in *fut6* leaf AGPs (Table 2). The fucosylation patterns of AGPs in wild-type and mutant lines, together with the expression patterns of *FUT4* and *FUT6*, clearly indicate that AGP fucosylation is attributable to both *FUT4* and *FUT6* genes in roots, but only to the *FUT4* gene in leaves. It would be interesting to investigate the role of *FUT4* in AGP fucosylation in stems and flowers, where the expression of *FUT4* was also abundant at least at a transcriptional level (Sarria *et al.*, 2001). The fact that *FUT3*, *FUT5*, and *FUT7*–*FUT10* were all expressed in leaves at different levels (Sarria *et al.*, 2001), but did not compensate for the lost function of *FUT4* in the *fut4* mutant, indicates that these genes might not be involved in AGP fucosylation and instead may fucosylate other non-AGP molecules. Alternatively, these genes may fucosylate AGPs, but only under certain non-standard conditions, which were not examined in this study. FUT4 and FUT6 were shown to differentially fucosylate AGPs, i.e. adding Fuc residues to different sites on AG side chains (Wu *et al.*, 2010). In support of this speculation, a structural study of AGPs from *Arabidopsis* leaves revealed the presence of at least three types of fucosylated oligosaccharides from the enzymatic digestion of a pool of AG side chains (Tryfona *et al.*, 2012). Given the extensive heterogeneity of polysaccharide structures present in AGP populations, some AG polysaccharides may lack Fuc, while others may contain Fuc added by one or both of the FUT enzymes. Moreover, FUT6 likely adds a terminal Fuc residue to AG polysaccharides that is particularly reactive with eel lectin, since the *fut6* mutant roots contained Fuc in AGPs but were not stained by eel lectin (Fig. 6 and Table 2). It is likely that epitopes recognized by eel lectin were synthesized by FUT6 and were abolished in *fut6* roots, whereas FUT4 still functioned in AGP fucosylation, but added the Fuc at a different site compared to FUT6; this second site is not recognized by the eel lectin.

AGPs are implicated in the regulation of root growth and root epidermal cell expansion, as treatment of *Arabidopsis* roots with β-Yariv reagent, which specifically aggregates some AGPs, results in a reduction of root length and epidermal cell length, as well as producing a ‘bulging’ phenotype of epidermal cells (Ding and Zhu, 1997; Willats and Knox, 1996). The *Arabidopsis mur1* mutant in the Columbia-0 background exhibited root phenotypes of shortened root length, which were attributed to shortened, swollen root hair cells presented in periodic zones of cells, interspersed by zones containing cells of normal length (van Hengel and Roberts, 2002). In addition, the length of the tip of *mur1* roots, measured from root cap to the first initiated root hair, has a 40% reduction in length compared to wild-type roots. The *mur1* mutant was deficient in synthesizing GDP-l-Fuc, the sugar donor substrate for the biosynthesis of Fuc-containing polymers (Bonin *et al.*, 1997). Considering that AGPs from *mur1* roots had a reduced Fuc content as compared to wild-type root and that eel lectin treatment of *Arabidopsis* plants phenocopies the *mur1* mutant in root, van Hengel and Roberts (2002) postulated that under-fucosylated AGPs were the cause of the *mur1* root phenotype. Consistent with the study of van Hengel and Roberts, Fuc residues were detected in AGPs from wild-type *Arabidopsis* roots (Table 2). However, the *fut4*/*fut6* double mutant, in which Fuc residues are not detected from root AGPs, did not show changes in the root phenotype (Table 2 and Fig. 4), indicating that the absence of Fuc residues in AGPs per se does not result in an abnormality of root growth. Besides AGPs, common Fuc-containing cell wall polymers include xyloglucan, pectin polymers, and N-glycans. Similar to the *fut4*/*fut6* double mutant reported here, mutants deficient in the fucosylation of xyloglucan or N-glycan had normal root growth, indicating that the fucosylation status of xyloglucan or N*-*glycan alone did not affect root morphogenesis (van Hengel and Roberts, 2002; Vanzin *et al.*, 2002). Thus, it cannot be excluded that the additive effects of under-fucosylation of multiple cell wall polymers resulted in the defects in root growth observed in the *mur1* mutant.

Compared to wild-type plants, *fut4*, *fut6*, and *fut4*/*fut6* mutants showed distinct glycome profiles for root and leaf cell wall extracts (Figs 7 and 8). Glycome profiling provides information about both the glycan epitope composition of cell walls, as well as how tightly those epitopes are bound into the wall matrix. To date, no antibody specifically directed at a fucosylated AGP epitope has been generated or characterized. The glycome profiles of the *fut* mutants examined here, not surprisingly, do not show the complete absence of a particular class of cell wall glycan epitopes. However, the glycome profiles of the *fut* mutants do show changes in the epitope compositions of individual extracts, suggesting that the mutations have affected the extractability of the glycan epitopes. For example, in the glycome profiles of cell wall extracts from roots (Fig. 7), many epitopes, including epitopes recognized by the NON-FUC XG, FUC XG, XYLAN-1, 3, HG BACKBONE, and RG-I BACKBONE groups of antibodies, showed increased signals in the carbonate extract from the *fut4* mutant, indicating that xyloglucan and pectin polymers carrying these epitopes become more extractable by carbonate in the *fut4* mutant root. On the other hand, the coupling of a decrease of signals in the more alkaline fractions with an increase of signals in the less alkaline fractions for RG-I/AG-, AG-4-, and XYLAN-3-reactive antibodies in the *fut* mutant root indicates that the corresponding polymers are more extractable in the mutants compared to the wild type. In contrast, antibodies in the NON-FUC XG and FUC XG groups showed decreased signals in the 1 and 4M KOH extracts for the *fut6* and *fut4*/*fut6* lines compared to the wild type, indicating decreased extractability of certain xyloglucan polymers in *fut6* and *fut4*/*fut6* roots. At a molecular level, AGPs have long been suggested to interact with other wall polymers or wall-modifying enzymes to modulate cell wall architectures (Roy *et al.*, 1998; Seifert and Roberts, 2007). Strong and specific binding between β-Yariv reagent and some AGPs implies that such AGPs may interact with β-glycan *in vivo* (Seifert and Roberts, 2007). Interactions between AGPs and pectic polymers were often suggested based on co-purification of the two polymers (Carpita, 1989; Immerzeel *et al.*, 2006; Iraki *et al.*, 1989). Binding between AGPs and pectin fractions was also shown in an *in vitro* binding experiment (Baldwin *et al.*, 1993). Recently, an *Arabidopsis* AGP protein encoded by At3g45230 was shown to bind to RG-I/HG and arabinoxylan through covalent bonds. Glycome profiling of *Arabidopsis* mutants defective in the At3g45230 gene had increased signals for pectin and xylan epitopes, suggesting increased extractability of the corresponding polymers (Tan *et al.*, 2013). In the current study, the changes of the extractability of wall polymers, as suggested by the variation of epitope abundance in different wall extracts, in essence reflect alterations in the interactions among cell wall polymers carrying the epitopes (i.e. AGPs, pectin, hemicellulose, and cellulose) in the *fut4*, *fut6*, and *fut4*/*fut6* mutants. Thus, Fuc residues on AGPs might play a critical role in mediating such interactions among wall polymers.

AGPs are involved in plant response to biotic and abiotic stresses (Gaspar *et al.*, 2004; Nam *et al.*, 1999; Shi *et al.*, 2003). Lamport *et al.* (2006) proposed the function of AGPs as plasticizer modulating the pectin network based on AGP release rate and distribution at cell surface of tobacco BY2 suspension cells subjected to salt stress. *fut4* and *fut6* mutants, with under-fucosylated root AGPs (Table 2), have normal root growth under standard conditions but significantly decreased root length compared to wild-type plants under salt treatment. The *fut4*/*fut6* double mutant, without any detectable Fuc in root AGPs (Table 2), has further reduced root length compared to *fut4* and *fut6* single mutants (Fig. 5, Fig. S6, and Table S2). It is likely that AGP fucosylation affects AGP function in modulating cell wall structure especially under stress conditions, although further studies are required to test this hypothesis. For example, examination by electron microscopy may elaborate structural changes in the mutant cell wall. As an initial attempt to look for potential roles of *FUT4* and *FUT6* under other non-standard conditions besides salt stress, expression profiles of the two genes were examined based on public microarray data, which show that the two genes are differentially affected by several treatments (Table S5).

In conclusion, the characterization of the *fut4*, *fut6*, and *fut4*/*fut6* mutants reported here further complements our previous biochemical study of FUT4 and FUT6 (Wu *et al.*, 2010), as it brings direct evidence that FUT4 fucosylates AGPs in leaves and that FUT4 and FUT6 fucosylate AGPs in roots of *Arabidopsis*. Non-fucosylated AGPs present in the mutants did not affect normal root growth, but nonetheless resulted in biochemical changes in overall cell wall structure in *fut4*, *fut6*, and *fut4*/*fut6* mutants as indicated by glycome profiling. Based on this profiling work it is likely that the Fuc residues present in AGPs function to regulate interactions between AGPs and other cell wall molecules and thus contribute to overall cell wall architecture. The current work also exemplifies the possibilities of refinement of cell wall structures by manipulation of a single or a few cell wall biosynthetic genes. Indeed, plants with such modified wall structures may display altered biomass recalcitrance and thus have the potential to enhance biofuel production.

## Supplementary material

Supplementary material is available at *JXB* online.

Supplementary Fig. S1. Real-time qPCR analysis of *FUT4* and *FUT6* mRNA level in root and leaf tissues of *Arabidopsis* wild-type and *fut4*, *fut6*, and *fut4*/*fut6* mutant plants.

Supplementary Fig. S2. Germination rate of *Arabidopsis fut4*, *fut6*, and *fut4*/*fut6* mutant and wild-type plants. Plant seeds were sown on MS plates containing 0.5% sucrose. Over 50 seeds were analysed for each line.

Supplementary Fig. S3. RNA transcript levels of the *FUT4* and *FUT6* genes in homozygous *fut4-2*, *fut6-2*, and wild-type seedlings. (A) Schematic diagrams of mutant *FUT4* and *FUT6* genes with T-DNA insertions. Black box, grey box, and white box represent exons, introns, and untranslated regions, respectively. (B) RT-PCR of total RNAs isolated from *Arabidopsis* seedlings 15 DAG. Positions of the RT-PCR primers are shown in (A) with arrows. *ACTIN* was used as a control for loading.

Supplementary Fig. S4. Real-time qPCR analysis of *FUT4* and *FUT6* mRNA levels in root and leaf tissues of *Arabidopsis* wild-type and *fut4-2* and *fut6-2* mutant plants.

Supplementary Fig. S5. Root growth of *Arabidopsis* wild-type and *fut4-2* and *fut6-2* mutant plants. Plants were grown on MS plates containing 0.5% sucrose. Root length was measured daily as a function of time from 3 to 10 DAG. Values shown were means of data from over 24 individual plants per line, with standard errors shown as error bars.

Supplementary Fig. S6. Root length measurements of *Arabidopsis fut4-2*, *fut6-2*, and wild-type plants grown under salt stress. *Arabidopsis* seeds were germinated on NaCl-free MS plates and then transferred to MS plates containing 150mM NaCl at 3 DAG. (A) Seedling images of wild-type and *fut4-2* and *fut6-2* mutant plants on the tenth day after transfer. Three representative seedlings of each plant line were photographed. Scale bar, 3cm. (B) Measurement of primary root length on the tenth day after transfer. Data and error bars represent mean±SD (*n*=31). Values annotated with different letters are significantly different (*P*<0.01; Tukey honestly significant difference test).

Supplementary Table S1. Sequence and annealing temperatures of the primers used in this work.

Supplementary Table S2. Statistic analysis for comparisons of root length measurements among wild-type and *fut4*, *fut4-2*, *fut6*, *fut6-2*, and *fut4*/*fut6* mutant plants grown under salt stress.

Supplementary Table S3. Expanded list of all plant cell wall glycan-directed monoclonal antibodies (mAbs) used in this study for glycome profiling (Figs 7 and 8).

Supplementary Table S4. Statistically significant differences in antibody binding between wild-type and mutant lines (*fut4*, *fut6*, and *fut4*/*fut6* mutants). The observed differences in the glycome profiles have been validated and confirmed by statistical analyses as explained in the Materials and methods and Results sections. Antibodies are sorted first by their glycan groups then by significant differences, with negative differences appearing first. The −log 10 of the *P* value is also shown. This is an indicator of significance. Higher numbers correlate to higher certainty that the differences noted were real.

Supplementary Table S5. Expression analysis of the *FUT4* and *FUT6* genes in response to biotic and abiotic stimuli based on microarray data from Genevestigator (https://www.genevestigator.com/gv/, last accessed 20 September 2013).

Supplementary Data

## Abbreviations:

AG: arabinogalactan
AGP: arabinogalactan-protein
ANOVA: analysis of variance
Ara: arabinose
CCRC: Complex Carbohydrate Research Center
DAG: days after germination
ELISA: enzyme-linked immunosorbent assay
Fuc: fucose
FUC XG: fucosylated xyloglucans
FUT: fucosyltransferase
Gal: galactose
GC-MS: gas chromatography-mass spectrometry
GT: glycosyltransferase
HG: homogalacturonan
HRGP: hydroxyproline-rich glycoprotein
MS: Murashige and Skoog
NON-FUC XG: non-fucosylated xyloglucans
qPCR: quantitative PCR
RG: rhamnogalacturonan.

## References

[CIT0001] Baldwin TC McCann MC Roberts K 1993 A novel hydroxyproline-deficient arabinogalactan protein secreted by suspension-cultured cells of *Daucus carota* (purification and partial characterization). Plant Physiology 103, 115–123 12231918 10.1104/pp.103.1.115 PMC158953

[CIT0002] Basu D Liang Y Liu X Himmeldirk K Faik A Kieliszewski M Held M Showalter AM 2013 Functional identification of a hydroxyproline-*O*-galactosyltransferase specific for arabinogalactan-protein biosynthesis in Arabidopsis. Journal of Biological Chemistry 288, 10132–10143 23430255 10.1074/jbc.M112.432609 PMC3617256

[CIT0003] Bonin CP Potter I Vanzin GF Reiter W-D 1997 The *MUR1* gene of *Arabidopsis thaliana* encodes an isoform of GDP-D-mannose-4,6-dehydratase, catalyzing the first step in the *de novo* synthesis of GDP-L-fucose. Proceedings of the National Academy of Sciences USA 94, 2085–2090 10.1073/pnas.94.5.2085 PMC20047 9050909

[CIT0004] Boyes DC Zayed AM Ascenzi R McCaskill AJ Hoffman NE Davis KR Gorlach J 2001 Growth stage-based phenotypic analysis of Arabidopsis: a model for high throughput functional genomics in plants. The Plant Cell 13, 1499–1510 11449047 10.1105/TPC.010011 PMC139543

[CIT0005] Cannon MC Terneus K Hall Q Tan L Wang Y Wegenhart BL Chen L Lamport DT Chen Y Kieliszewski MJ 2008 Self-assembly of the plant cell wall requires an extensin scaffold. Proceedings of the National Academy of Sciences USA 105, 2226–2231 10.1073/pnas.0711980105 PMC2538902 18256186

[CIT0006] Carpita N McCann M 2000 The cell wall. In Buchanan BB Gruissem W Jones R, eds, Biochemistry and Molecular Biology of Plants. American Society of Plant Physiologists, Rockville, MD, pp 52–109

[CIT0007] Carpita NC 1989 Pectic polysaccharides of maize coleoptiles and proso millet cells in liquid culture. Phytochemistry 28, 121–125

[CIT0008] Carpita NC Gibeaut DM 1993 Structural models of primary-cell walls in flowering plants - consistency of molecular-structure with the physical-properties of the walls during growth. The Plant Journal 3, 1–30 8401598 10.1111/j.1365-313x.1993.tb00007.x

[CIT0009] Ding L Zhu JK 1997 A role for arabinogalactan-proteins in root epidermal cell expansion. Planta 203, 289–294 9431677 10.1007/s004250050194

[CIT0010] DuBois M Gilles KA Hamilton JK Rebers PA Smith F 1956 Colorimetric method for determination of sugars and related substances. Analytical Chemistry 28, 350–356

[CIT0011] Ellis M Egelund J Schultz CJ Bacic A 2010 Arabinogalactan-proteins: key regulators at the cell surface? Plant Physiology 153, 403–419 20388666 10.1104/pp.110.156000 PMC2879789

[CIT0012] Faik A Bar-Peled M DeRocher AE Zeng WQ Perrin RM Wilkerson C Raikhel NV Keegstra K 2000 Biochemical characterization and molecular cloning of an alpha-1,2-fucosyltransferase that catalyzes the last step of cell wall xyloglucan biosynthesis in pea. Journal of Biological Chemistry 275, 15082–15089 10747946 10.1074/jbc.M000677200

[CIT0013] Fry SC 1988 The Growing Plant Cell Wall: Chemical and Metabolic Analysis. Longman Scientific & Technical, Essex

[CIT0014] Gaspar YM Nam J Schultz CJ Lee L-Y Gilson PR Gelvin SB Bacic A 2004 Characterization of the Arabidopsis lysine-rich arabinogalactan-protein ATAGP17 mutant (*rat1*) that results in a decreased efficiency of *Agrobacterium* transformation. Plant Physiology 135, 2162–2171 15286287 10.1104/pp.104.045542 PMC520787

[CIT0015] Geshi N Johansen JN Dilokpimol A 2013 A galactosyltransferase acting on arabinogalactan protein glycans is essential for embryo development in Arabidopsis. The Plant Journal,10.1111/tpj.12281 10.1111/tpj.12281 23837821

[CIT0016] Gibeaut DM Hulett J Cramer GR Seemann JR 1997 Maximal biomass of *Arabidopsis thaliana* using a simple, low-maintenance hydroponic method and favorable environmental conditions. Plant Physiology 115, 317–319 9342857 10.1104/pp.115.2.317 PMC158488

[CIT0017] Gille S Sharma V Baidoo EEK Keasling JD Scheller HV Pauly M 2013 Arabinosylation of a Yariv-precipitable cell wall polymer impacts plant growth as exemplified by the *Arabidopsis* glycosyltransferase mutant *ray1* . Molecular Plant 6, 1369–1372 23396039 10.1093/mp/sst029

[CIT0018] Gorshkova TA Wyatt SE Salnikov VV Gibeaut DM Ibragimov MR Lozovaya VV Carpita NC 1996 Cell-wall polysaccharides of developing flax plants. Plant Physiology 110, 721–729 12226214 10.1104/pp.110.3.721 PMC157770

[CIT0019] Günter EA Popeĭko OV Ovodov Iu S 2004 Isolation of polysaccharides from callus culture of *Lemna minor* L. Applied Biochemistry and Microbiology 40, 80–83 15029707

[CIT0020] Hashimoto Y 2000 Structure and biosynthesis of L-fucosylated arabinogalactan-proteins in cruciferous plants. In Nothnagel EA Bacic A Clarke AE, eds, Cell and Developmental Biology of Arabinogalactan-Proteins. Kluwer Academic/Plenum Publishers, New York, pp 51–60

[CIT0021] Hayashi T Maclachlan G 1984 Glycolipids and glycoproteins formed from UDP-galactose by pea membranes. Phytochemistry 23, 487–492

[CIT0022] Immerzeel P Eppink MM De Vries SC Schols HA Voragen AGJ 2006 Carrot arabinogalactan proteins are interlinked with pectins. Physiologia Plantarum 128, 18–28

[CIT0023] Iraki NM Singh N Bressan RA Carpita NC 1989 Cell-walls of tobacco cells and changes in composition associated with reduced growth upon adaptation to water and saline stress. Plant Physiology 91, 48–53 16667041 10.1104/pp.91.1.48 PMC1061950

[CIT0024] Lamport DT Kieliszewski MJ Showalter AM 2006 Salt stress upregulates periplasmic arabinogalactan proteins: using salt stress to analyse AGP function. New Phytologist 169, 479–492 16411951 10.1111/j.1469-8137.2005.01591.x

[CIT0025] Lamport DTA Kieliszewski MJ Chen Y Cannon MC 2011 Role of the extensin superfamily in primary cell wall architecture. Plant Physiology 156, 11–19 21415277 10.1104/pp.110.169011 PMC3091064

[CIT0026] Liang Y Faik A Kieliszewski M Tan L Xu WL Showalter AM 2010 Identification and characterization of in vitro galactosyltransferase activities involved in arabinogalactan-protein glycosylation in tobacco and Arabidopsis. Plant Physiology 154, 632–642 20671109 10.1104/pp.110.160051 PMC2949012

[CIT0027] Mascara T Fincher GB 1982 Biosynthesis of arabinogalactan-protein in *Lolium multiflorum* (ryegrass) endosperm cells. 2. *In vitro* incorporation of galactosyl residues from UDP-galactose into polymeric products. Australian Journal of Plant Physiology 9, 31–45

[CIT0028] Masuko T Minami A Iwasaki N Majima T Nishimura S-I Lee YC 2005 Carbohydrate analysis by a phenol-sulfuric acid method in microplate format. Analytical Biochemistry 339, 69–72 15766712 10.1016/j.ab.2004.12.001

[CIT0029] Memelink J Swords KMM Dekam RJ Schilperoort RA Hoge JHC Staehelin LA 1993 Structure and regulation of tobacco extensin. The Plant Journal 4, 1011–1022 7506607 10.1046/j.1365-313x.1993.04061011.x

[CIT0030] Misawa H Tsumuraya Y Kaneko Y Hashimoto Y 1996 Alpha-L-fucosyltransferases from radish primary roots. Plant Physiology 110, 665–673 8742340 10.1104/pp.110.2.665 PMC157763

[CIT0031] Nam J Mysore KS Zheng C Knue MK Matthysse AG Gelvin SB 1999 Identification of T-DNA tagged Arabidopsis mutants that are resistant to transformation by *Agrobacterium* . Molecular and General Genetics 261, 429–438 10323222 10.1007/s004380050985

[CIT0032] Nothnagel EA 1997 Proteoglycans and related components in plant cells. International Review of Cytology. A Survey of Cell Biology. 174, 195–291 10.1016/s0074-7696(08)62118-x 9161008

[CIT0033] Oka T Saito F Shimma YI Yoko-O T Nomura Y Matsuoka K Jigami Y 2010 Characterization of endoplasmic reticulum-localized UDP-D-galactose: hydroxyproline *O*-galactosyltransferase using synthetic peptide substrates in *Arabidopsis* . Plant Physiology 152, 332–340 19923238 10.1104/pp.109.146266 PMC2799367

[CIT0034] Pattathil S Avci U Baldwin D 2010 A comprehensive toolkit of plant cell wall glycan-directed monoclonal antibodies. Plant Physiology 153, 514–525 20363856 10.1104/pp.109.151985 PMC2879786

[CIT0035] Pattathil S Avci U Miller JS Hahn MG 2012 Immunological approaches to plant cell wall and biomass characterization: glycome profiling. In Himmel M, ed, Biomass Conversion: Methods and Protocols, Methods in Molecular Biology, Vol 908 Springer Science & Business Media, New York, pp 61–72 10.1007/978-1-61779-956-3_6 22843389

[CIT0036] Perrin RM DeRocher AE Bar-Peled M Zeng WQ Norambuena L Orellana A Raikhel NV Keegstra K 1999 Xyloglucan fucosyltransferase, an enzyme involved in plant cell wall biosynthesis. Science 284, 1976–1979 10373113 10.1126/science.284.5422.1976

[CIT0037] Ponce MR Quesada V Micol JL 1998 Rapid discrimination of sequences flanking and within T-DNA insertions in the Arabidopsis genome. The Plant Journal 14, 497–501 9670564 10.1046/j.1365-313x.1998.00146.x

[CIT0038] Puhlmann J Bucheli E Swain MJ Dunning N Albersheim P Darvill AG Hahn MG 1994 Generation of monoclonal-antibodies against plant cell-wall polysaccharides. I. Characterization of a monoclonal-antibody to a terminal alpha-(1→2)-linked fucosyl-containing epitope. Plant Physiology 104, 699–710 7512736 10.1104/pp.104.2.699 PMC159249

[CIT0039] Reiter WD Chapple C Somerville CR 1997 Mutants of *Arabidopsis thaliana* with altered cell wall polysaccharide composition. The Plant Journal 12, 335–345 9301086 10.1046/j.1365-313x.1997.12020335.x

[CIT0040] Roy S Jauh GY Hepler PK Lord EM 1998 Effects of Yariv phenylglycoside on cell wall assembly in the lily pollen tube. Planta 204, 450–458 9684368 10.1007/s004250050279

[CIT0041] Sarria R Wagner TA O’Neill MA Faik A Wilkerson CG Keegstra K Raikhel NV 2001 Characterization of a family of Arabidopsis genes related to xyloglucan fucosyltransferase1. Plant Physiology 127, 1595–1606 11743104 PMC133564

[CIT0042] Schibeci A Pnjak A Fincher GB 1984 Biosynthesis of arabinogalactan-protein in *Lolium multiflorum* (Italian ryegrass) endosperm cells - subcellular-distribution of galactosyltransferases. Biochemical Journal 218, 633–636 6424663 10.1042/bj2180633 PMC1153383

[CIT0043] Schultz CJ Johnson KL Currie G Bacic A 2000 The classical arabinogalactan protein gene gamily of Arabidopsis. The Plant Cell 12, 1751–1768 11006345 10.1105/tpc.12.9.1751 PMC149083

[CIT0044] Seifert GJ Roberts K 2007 The biology of arabinogalactan proteins. Annual Review of Plant Biology 58, 137–161 10.1146/annurev.arplant.58.032806.103801 17201686

[CIT0045] Shi HZ Kim Y Guo Y Stevenson B Zhu JK 2003 The Arabidopsis SOS5 locus encodes a putative cell surface adhesion protein and is required for normal cell expansion. The Plant Cell 15, 19–32 12509519 10.1105/tpc.007872 PMC143448

[CIT0046] Showalter AM 1993 Structure and function of plant cell wall proteins. The Plant Cell 5, 9–23 8439747 10.1105/tpc.5.1.9 PMC160246

[CIT0047] Showalter AM 2001 Arabinogalactan-proteins: structure, expression and function. Cellular and Molecular Life Sciences 58, 1399–1417 11693522 10.1007/PL00000784 PMC11337269

[CIT0048] Showalter AM Keppler B Lichtenberg J Gu DZ Welch LR 2010 A bioinformatics approach to the identification, classification, and analysis of hydroxyproline-rich glycoproteins. Plant Physiology 153, 485–513 20395450 10.1104/pp.110.156554 PMC2879790

[CIT0049] Shpak E Barbar E Leykam JF Kieliszewski MJ 2001 Contiguous hydroxyproline residues direct hydroxyproline arabinosylation in *Nicotiana tabacum* . Journal of Biological Chemistry 276, 11272–11278 11154705 10.1074/jbc.M011323200

[CIT0050] Springer GF Desai PR 1971 Monosaccharides as specific precipitinogens of eel antihuman blood-group H(O) antibody. Biochemistry 10, 3749–3761 4999530 10.1021/bi00796a017

[CIT0051] Tan L Eberhard S Pattathil S 2013 An Arabidopsis cell wall proteoglycan consists of pectin and arabinoxylan covalently linked to an arabinogalactan protein. The Plant Cell 25, 270–287 23371948 10.1105/tpc.112.107334 PMC3584541

[CIT0052] Tan L Qiu F Lamport DT Kieliszewski MJ 2004 Structure of a hydroxyproline (Hyp)-arabinogalactan polysaccharide from repetitive Ala-Hyp expressed in transgenic *Nicotiana tabacum* . Journal of Biological Chemistry 279, 13156–13165 14724279 10.1074/jbc.M311864200

[CIT0053] Tryfona T Liang H-C Kotake T Tsumuraya Y Stephens E Dupree P 2012 Structural characterisation of Arabidopsis leaf arabinogalactan polysaccharides. Plant Physiology 160, 653–666 22891237 10.1104/pp.112.202309 PMC3461546

[CIT0054] Tsumuraya Y Hashimoto Y Yamamoto S 1987 An L-arabino-D-galactan and an L-arabino-D-galactan-containing proteoglycan from radish (*Raphanus sativus*) seeds. Carbohydrate Research 161, 113–126

[CIT0055] Tsumuraya Y Hashimoto Y Yamamoto S Shibuya N 1984 Structure of L-arabino-D-galactan-containing glycoproteins from radish leaves. Carbohydrate Research 134, 215–228

[CIT0056] Tsumuraya Y Ogura K Hashimoto Y Mukoyama H Yamamoto S 1988 Arabinogalactan-proteins from primary and mature roots of radish (*Raphanus sativus* L.). Plant Physiology 86, 155–160 16665859 10.1104/pp.86.1.155 PMC1054447

[CIT0057] van Hengel AJ Roberts K 2002 Fucosylated arabinogalactan-proteins are required for full root cell elongation in Arabidopsis. The Plant Journal 32, 105–113 12366804 10.1046/j.1365-313x.2002.01406.x

[CIT0058] Vanzin GF Madson M Carpita NC Raikhel NV Keegstra K Reiter WD 2002 The *mur2* mutant of *Arabidopsis thaliana* lacks fucosylated xyloglucan because of a lesion in fucosyltransferase AtFUT1. Proceedings of the National Academy of Sciences USA 99, 3340–3345 10.1073/pnas.052450699 PMC122520 11854459

[CIT0059] Watkins WM Morgan WT 1952 Neutralization of the anti-H agglutinin in eel serum by simple sugars. Nature. 169, 825–826 14941057 10.1038/169825a0

[CIT0060] Wei G Shirsat AH 2006 Extensin over-expression in Arabidopsis limits pathogen invasiveness. Molecular Plant Pathology 7, 579–592 20507471 10.1111/j.1364-3703.2006.00363.x

[CIT0061] Willats WGT Knox JP 1996 A role for arabinogalactan-proteins in plant cell expansion: evidence from studies on the interaction of beta-glucosyl Yariv reagent with seedlings of *Arabidopsis thaliana* . The Plant Journal 9, 919–925 8696368 10.1046/j.1365-313x.1996.9060919.x

[CIT0062] Wu YY Williams M Bernard S Driouich A Showalter AM Faik A 2010 Functional identification of two nonredundant Arabidopsis α(1,2)fucosyltransferases specific to arabinogalactan proteins. Journal of Biological Chemistry 285, 13638–13645 20194500 10.1074/jbc.M110.102715 PMC2859526

[CIT0063] Xu JF Tan L Lamport DTA Showalter AM Kieliszewski MJ 2008 The *O*-Hyp glycosylation code in tobacco and *Arabidopsis* and a proposed role of Hyp-glycans in secretion. Phytochemistry 69, 1631–1640 18367218 10.1016/j.phytochem.2008.02.006

[CIT0064] Yong WD Link B O’Malley R 2005 Genomics of plant cell wall biogenesis. Planta 221, 747–751 15981004 10.1007/s00425-005-1563-z

[CIT0065] Zhan X Wang B Li H Liu R Kalia RK Zhu J-K Chinnusamy V 2012 Arabidopsis proline-rich protein important for development and abiotic stress tolerance is involved in microRNA biogenesis. Proceedings of the National Academy of Sciences USA 109, 18198–18203 10.1073/pnas.1216199109 PMC3497810 23071326

[CIT0066] Zhu S-Y Yu X-C Wang X-J 2007 Two calcium-dependent protein kinases, CPK4 and CPK11, regulate abscisic acid signal transduction in Arabidopsis. The Plant Cell 19, 3019–3036 17921317 10.1105/tpc.107.050666 PMC2174700

