# Supplementary material for: Biochemical and physiological characterization of fut4 and fut6 mutants defective in arabinogalactan-protein fucosylation in Arabidopsis
Source: J Exp Bot. 2013 Oct 14;64(18):5537–51. doi: 10.1093/jxb/ert321 (PMC3871811; doi:10.1093/jxb/ert321)
Supplement: Supplementary Data [file supp_64_18_5537__index.html]

Biochemical and physiological characterization of fut4 and fut6 mutants defective in arabinogalactan-protein fucosylation in Arabidopsis — Biochemical and physiological characterization of fut4 and fut6 mutants defective in arabinogalactan-protein fucosylation in Arabidopsis — Supplementary Data 

# Biochemical and physiological characterization of *fut4* and *fut6* mutants defective in arabinogalactan-protein fucosylation in *Arabidopsis*

## Supplementary Data

Data files

**Files in this Data Supplement:**

- Supplementary Data - Supplementary Data
- Supplementary Data - Supplementary Data
- Supplementary Data - Supplementary Data
