# Supplementary material for: Biochemical and physiological characterization of fut4 and fut6 mutants defective in arabinogalactan-protein fucosylation in Arabidopsis
Source: J Exp Bot. 2013 Oct 14;64(18):5537–51. doi: 10.1093/jxb/ert321 (PMC3871811; doi:10.1093/jxb/ert321)
Supplement: Supplementary Data [file supp_ert321_jexbot098897_file001.pdf]

Figure S1

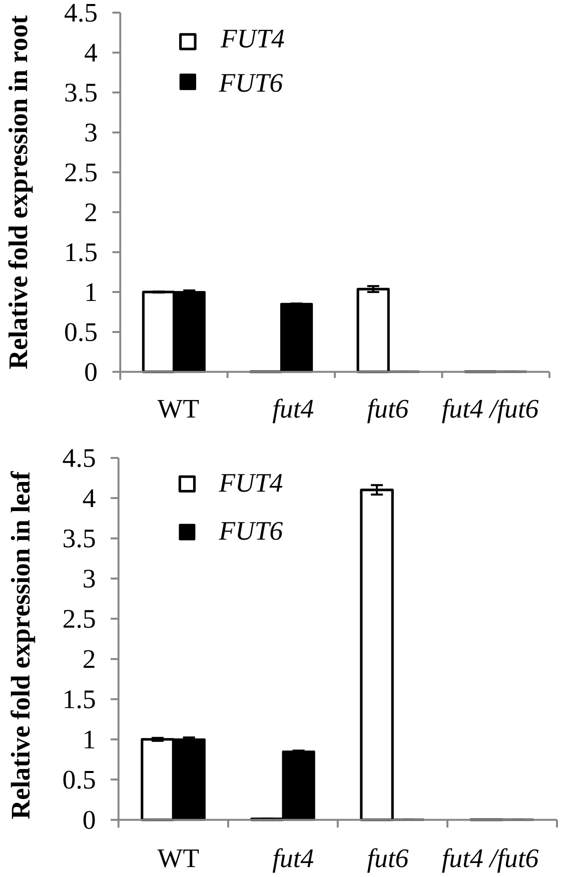

Figure S1. RT-qPCR analysis of *FUT4* and *FUT6* mRNA level in root and leaf tissues of Arabidopsis wild type and *fut4*, *fut6* and *fut4/fut6* double mutant plants.

Figure S2

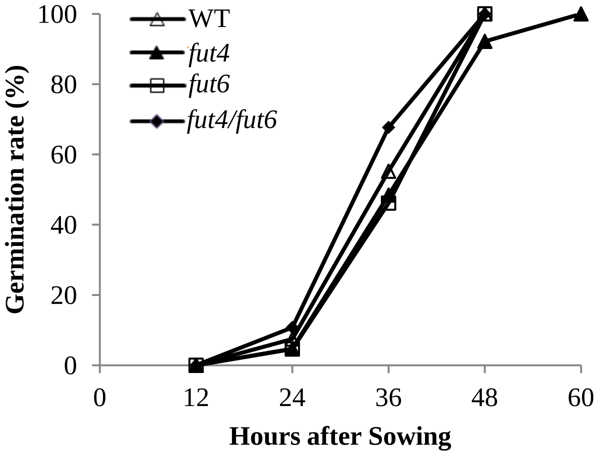

Figure S2. Germination rate of Arabidopsis *fut4*, *fut6* and *fut4/fut6* double mutant and wild type plants. Plant seeds were sown on MS plates containing 0.5% sucrose. Over 50 seeds were analyzed for each line.

Figure S3

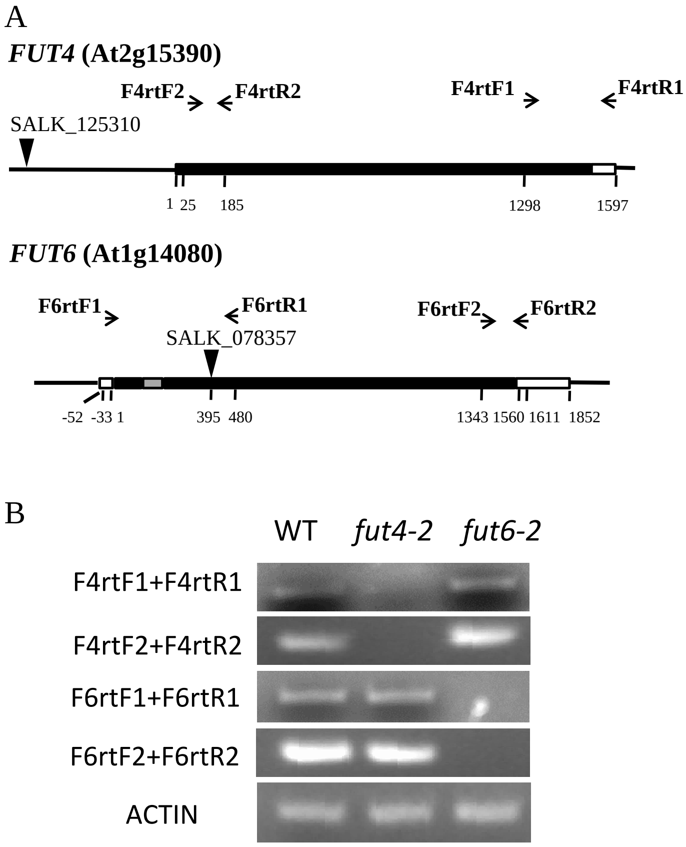

Figure S3. RNA transcript levels of *FUT4* and *FUT6* genes in homozygous *fut4-2*, *fut6-2* mutant and wild type seedlings. A, Schematic diagrams of mutant *FUT4* and *FUT6* genes with T-DNA insertions. Black box, grey box and white box represent exons, introns and untranslated regions, respectively. B, RT-PCR of total RNAs isolated from Arabidopsis seedlings 15 DAG. Positions of the RT-PCR primers are shown in A with arrows. *ACTIN* was used as a control for loading.

Figure S4

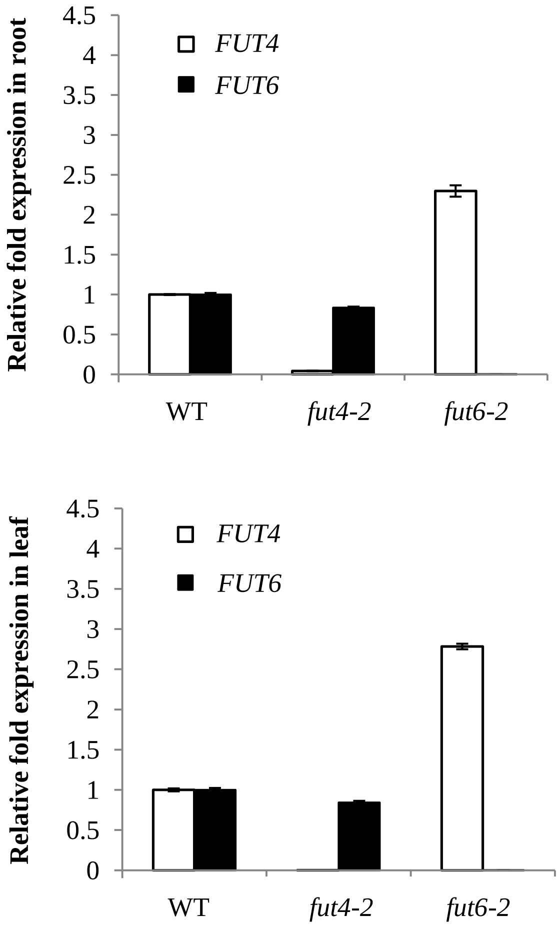

Figure S4. RT-qPCR analysis of *FUT4* and *FUT6* mRNA level in root and leaf tissues of Arabidopsis wild type and *fut4-2* and *fut6-2* mutant plants.

Figure S5

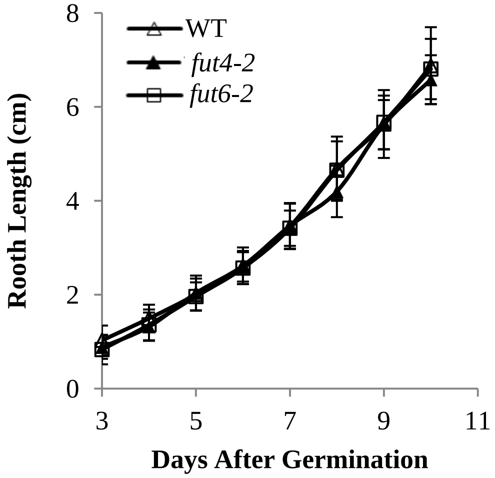

Figure S5. Root growth of Arabidopsis wild type, *fut4-2*, *fut6-2* mutant plants. Plants were grown on MS plates containing 0.5% sucrose. Root length was measured daily as a function of time from 3-10 DAG. Values shown were averages of data from over 24 individual plants per line, with standard errors shown as error bars.

Figure S6

A

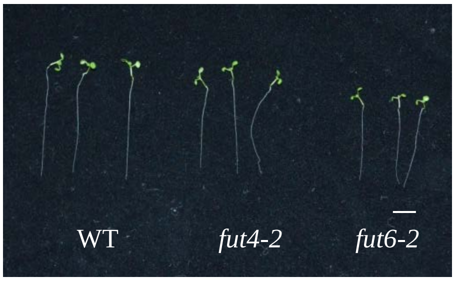

B

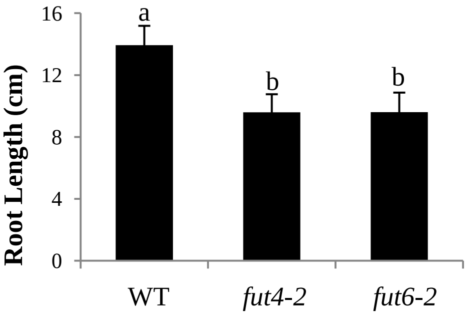

Figure S6. Root length measurement of Arabidopsis *fut4-2*, *fut6-2* and wild type plants grown under salt stress. Arabidopsis seeds were germinated on NaCl-free MS plates and then transferred to MS plates containing 150 mM NaCl on the 3 DAG. A, Seedling images of wild type, *fut4-2* and *fut6-2* mutant plants on the 10th day after transfer. Three representative seedlings of each plant line were photographed. Scale bar, 3 cm. B, Measurement of primary root length on the 10th day after transfer. Data and error bars represent mean $\pm$ SD (n = 31). Values annotated with different letters are significantly different ($P < 0.01$; Tukey honestly significant difference test).

Table S1

Table S1. Sequence and annealing temperatures of the primers used in this work

Purpose	Name	Primer Sequences (5'→3')	Annealing
Absence of insertion of *FUT4*	F4LP	CCATGTAGTTACATTCCCAACCG	54° C
	F4RP	CCACGTCGATGGAGCCTTGTTT	
Presence of insertion 1 in SAIL_284_B05	LB3	TAGCATCTGAATTTCATAACCAATCTCGATACAC	49° C
	F4RP	GAACATGTTTTCAGAGCGAGC	
Presence of insertion 2 in SAIL_284_B05	LB3	TAGCATCTGAATTTCATAACCAATCTCGATACAC	54° C
	F4LP	CCATGTAGTTACATTCCCAACCG	
Absence of insertion of *FUT6*	F6LP	CACATCTTTCAGATCTCCAGCG	55° C
	F6RP	CTTTCTTGTAAGCATCCGTGC	
Presence of insertion 1 in SALK_099500	LBa1	TGGTTCACGTAGTGGGCCATCG	54° C
	F6RP	CTTTCTTGTAAGCATCCGTGC	
Presence of insertion 2 in SALK_099500	LBa1	TGGTTCACGTAGTGGGCCATCG	54° C
	F6LP	TGTTTTGCTAGCAACGAAAGC	
RT -PCR for *FUT4* 3' end primers	F4rtF1	TGGAGGATTAAAGCCATGGTTAC	52° C
	F4rtR1	AATGAAAAAGGAAGATATATAACA	
RT -PCR for *FUT6* 5' end primers	F6rtF1	TCCAGCGAAGTTTTCAGAGC	52° C
	F6rtR1	CGCAACCCACACAATGTATC	
RT -PCR for *FUT4* 5' end primers	F4rtF2	GCGAAGTTATCAAGGGTTTGGG	58° C
	F4rtR2	CTTGTATCGCAAGCCTTCACC	
RT -PCR for *FUT6* 3' end primers	F6rtF2	TGGAGGATTAAAGCCATGGTTAC	58° C
	F6rtR2	GGATTGTGAGTTATTGAAACAGAGA	
RT -PCR for *ACTIN*	Actin F	GTCGTACTACCGGTATTGTGCTCG	58° C
	Actin R	CGGCGATTCCAGGGAACATTGTGG	
Ubiquitin 10 for RT-qPCR	Ubi F	GTCGACCCTTCACTTGGTGT	58° C
	Ubi R	ATCCTCAAGCTGCTTTCCAG	

Table S2

Table S2. Statistic analysis for comparisons of root length measurements among wild type and *fut4, fut4-2, fut6, fut6-2, fut4/fut6* mutant plants grown under salt stress.

P [a]	WT	*fut4*	*fut4-2*	*fut6*	*fut6-2*	*fut4/fut6*
WT	-	$<10^{-4}$	$<10^{-4}$	$<10^{-4}$	$<10^{-4}$	$<10^{-4}$
fut4	$<10^{-4}$	-	0.9462	0.9909	0.9382	0.0013
fut4-2	$<10^{-4}$	0.9462	-	0.9997	1.0000	$<10^{-4}$
fut6	$<10^{-4}$	0.9909	0.9997	-	0.9995	$<10^{-4}$
fut6-2	$<10^{-4}$	0.9382	1.0000	0.9995	-	$<10^{-4}$
fut4/fut6	$<10^{-4}$	0.0013	$<10^{-4}$	$<10^{-4}$	$<10^{-4}$	-

[a]One-way ANOVA was performed using JMP software and showed significant difference ($P<0.0001$) in root lengths among wild type and mutant lines grown under salt stress (150 mM NaCl). Significant levels of differences in root lengths were subsequently analyzed by Tukey honestly significant difference test as presented in this table. For root measurement data and plant growth conditions see Fig. 5 and Fig. S6.

Table S3

Table S3. Expanded list of all plant cell wall glycan-directed monoclonal antibodies (mAbs) used in this study for glycome profiling (Fig. 7 and Fig. 8).

Glycan Group Recognized	mAb Name[a]
Non-Fucosylated Xyloglucan	CCRC-M54
	CCRC-M48
	CCRC-M49
	CCRC-M96
	CCRC-M50
	CCRC-M51
	CCRC-M53
	CCRC-M100
	CCRC-M103
	CCRC-M58
	CCRC-M86
	CCRC-M55
	CCRC-M52
	CCRC-M99
	CCRC-M95
	CCRC-M101
	CCRC-M104
	CCRC-M89
	CCRC-M93
	CCRC-M87
	CCRC-M88
	CCRC-M57
	CCRC-M90
Fucosylated Xyloglucan	CCRC-M102
	CCRC-M39
	CCRC-M106
	CCRC-M84
	CCRC-M1
Xylan 1/XG	CCRC-M111
	CCRC-M108
	CCRC-M109

Xylan 2	CCRC-M119
	CCRC-M115
	CCRC-M110
	CCRC-M105
	CCRC-M117
	CCRC-M113
	CCRC-M120
	CCRC-M118
	CCRC-M116
	CCRC-M114
	CCRC-M154
	CCRC-M150
Xylan 3	CCRC-M160
	CCRC-M137
	CCRC-M152
	CCRC-M149
	CCRC-M144
	CCRC-M146
	CCRC-M145
	CCRC-M155
Xylan 4	CCRC-M153
	CCRC-M151
	CCRC-M148
	CCRC-M140
	CCRC-M139
	CCRC-M138
Seed Galactomannan	CCRC-M75
	CCRC-M70
	CCRC-M74
Homogalacturonan Backbone	CCRC-M131
	CCRC-M38
	JIM5
	JIM136
	JIM7
	CCRC-M34
Rhamnogalacturonan I Backbone	CCRC-M69
	CCRC-M35
	CCRC-M36

	CCRC-M14<br>CCRC-M129<br>CCRC-M72
Linseed Mucilage Rhamnogalacturonan I	CCRC-M40
Physcomitrella Pectin	CCRC-M98<br>CCRC-M94
Rhamnogalacturonan IA	CCRC-M5<br>CCRC-M2
Rhamnogalacturonan IB	CCRC-M23<br>CCRC-M17<br>CCRC-M19<br>CCRC-M18<br>CCRC-M56<br>CCRC-M16
Rhamnogalacturonan IC	JIM137<br>JIM101<br>CCRC-M61<br>CCRC-M30
Rhamnogalacturonan I Arabinogalactan	CCRC-M60<br>CCRC-M41<br>CCRC-M80<br>CCRC-M79<br>CCRC-M44<br>CCRC-M33<br>CCRC-M32<br>CCRC-M13<br>CCRC-M42<br>CCRC-M24<br>CCRC-M12<br>CCRC-M7<br>CCRC-M77<br>CCRC-M25<br>CCRC-M9<br>CCRC-M128<br>CCRC-M126<br>CCRC-M134<br>CCRC-M125

	CCRC-M123<br>CCRC-M122<br>CCRC-M121<br>CCRC-M112<br>CCRC-M21<br>JIM131<br>CCRC-M22<br>JIM132<br>JIM1<br>CCRC-M15<br>CCRC-M8<br>MH4.3E5<br>JIM16
Arabinogalactan 1	JIM93<br>JIM94<br>JIM11<br>MAC204<br>JIM20
Arabinogalactan 2	JIM14<br>MAC207<br>JIM19<br>JIM12<br>CCRC-M133<br>CCRC-M107
Arabinogalactan 3	JIM4<br>CCRC-M31<br>JIM17<br>CCRC-M26<br>JIM15<br>JIM8<br>CCRC-M85<br>CCRC-M81<br>MAC266<br>PN 16.4B4
Arabinogalactan 4	JIM133<br>JIM13<br>CCRC-M92<br>CCRC-M91

	CCRC-M78
Unidentified	MAC265<br>CCRC-M97

[a]The majority of listings link to the Wall*Mab*DB plant cell wall monoclonal antibody database (http://www.wallmabdb.net) that provides detailed descriptions of each mAb, including immunogen, antibody isotype, epitope structure (to the extent known), supplier information, and related literature citations.
